# Recent Advances in One-Pot Multicomponent Reactions for the Synthesis of Substituted Quinazolin-4(3*H*)-ones

**DOI:** 10.3390/molecules30183729

**Published:** 2025-09-13

**Authors:** Zbigniew Malinowski

**Affiliations:** Department of Organic Chemistry, Faculty of Chemistry, University of Lodz, Tamka 12, 91-403 Lodz, Poland; zbigniew.malinowski@chemia.uni.lodz.pl

**Keywords:** quinazolinones, multicomponent reactions, one-pot reactions

## Abstract

Quinazolin-4(3*H*)-ones are nitrogen heterocycles that have attracted considerable interest over many years due to their important biological and pharmacological properties. It has been shown that quinazolinone derivatives exhibit, e.g., analgesic, anti-inflammatory, antibacterial, anticonvulsant, antifungal, and antitumor activities. Some of these compounds have found applications in medicine; for instance, Zydelig (Idelalisib) has been approved for the treatment of several types of blood cancers. Furthermore, the quinazolinone skeleton is an important structural moiety present in many naturally occurring alkaloids, such as Febrifugine, a potent anti-malarial agent. To date, numerous synthetic methods have been developed for the synthesis of quinazolinone derivatives. Among them, multicomponent reactions (MCRs) have emerged as a powerful tool, allowing for the rapid and straightforward construction of the quinazolinone scaffold from readily available substrates. This review article presents a concise overview of selected strategies for synthesizing quinazolinone frameworks via one-pot MCRs. The reported methods are categorized into three main groups: metal-catalyzed reactions; isatoic-anhydride-based strategies, utilizing isatoic anhydride as a key starting material, and alternative approaches involving, among others, the utilization of *N*-(2-aminobenzoyl)benzotriazoles or aryldiazonium salts as efficient building materials.

## 1. Introduction

**Figure 1:**
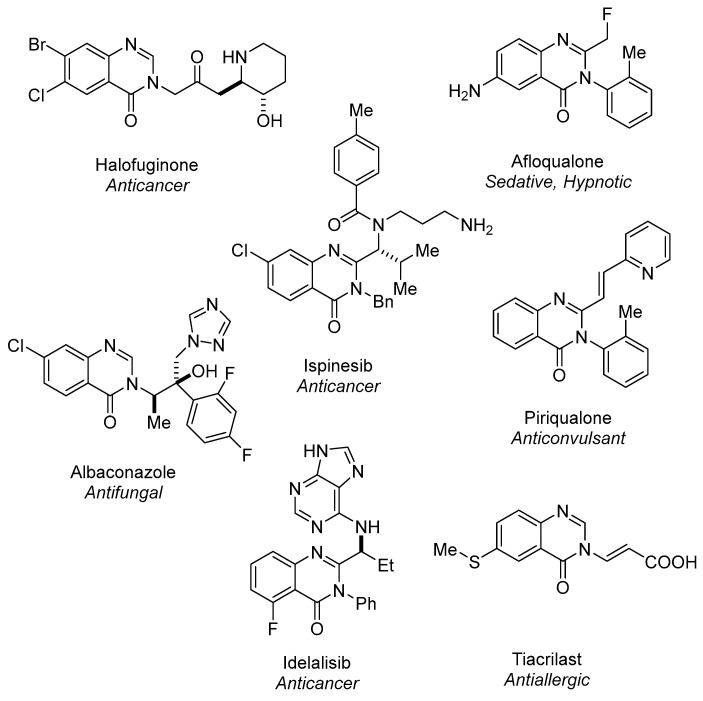
Quinazolinone medications [10].

Quinazolin-4(3*H*)-one and its derivatives are a group of pharmacologically active nitrogen-containing heterocycles that are of great interest in medicinal chemistry [1,2]. Quinazolin-4(3*H*)-one, in particular, is an important building block in the synthesis of various bioactive substances. Compounds containing the quinazolinone moiety demonstrate a broad spectrum of biological activities, including analgesic [3], anti-inflammatory [4], antibacterial [5], anticonvulsant [6], antifungal [7], and, particularly, antitumor effects [8]. Additionally, some of the quinazolinone derivatives have been identified as inhibitors of tubulin polymerization [9]. The fact that several marketed drugs are also based on the quinazolinone structure further underscores the importance and potential of this compound class as effective therapeutic agents [10] (Figure 1).

**Figure 2:**
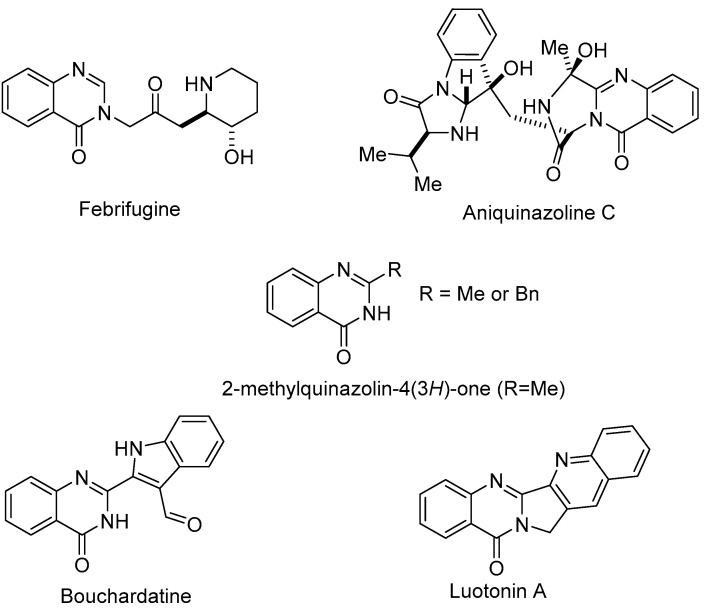
Naturally occurring quinazolinone alkaloids.

The quinazolinone skeleton is also widely found in many naturally occurring alkaloids isolated from various plants and microorganisms (Figure 2). Examples include Febrifugine, a potent anti-malarial agent isolated from the roots and leaves of the Asian plant *Dichroa febrifuga* [11], Aniquinazolines A-D, derived from the marine endophytic fungus *Aspergillus nidulans*, isolated from the leaves of *Rhizophora stylosa* [12], and 2-methylquinazolin-4(3*H*)-one and 2-benzylquinazolin-4(3*H*)-one, both obtained from *Bacillus cereus* [13]. Other examples include Bouchardatine, a *β*-indoloquinazolinone alkaloid found in *Bouchardatia neurococca* [14], and Luotonin A, which was isolated from *Peganum nigellastrum* Bunge [15].

Due to the wide range of biological activities exhibited by quinazolinone derivatives and the importance of these compounds as potential therapeutic candidates, numerous synthetic methods have been developed for their preparation. Classical methodologies for constructing the quinazolinone ring system, based on the Niementowski reaction and the condensation of anthranilic acid derivatives with carbonyl compounds, carboxylic acids, aryl halides, or ortho esters and amines, often require the use of hazardous chemicals, strong oxidants, and either high temperatures or long reaction times under specific reaction conditions [16]. Therefore, the development of novel, efficient, and, above all, environmentally friendly strategies for constructing the quinazolin-4(3*H*)-one scaffold remains a significant challenge that continues to attract considerable attention. Recent advances have introduced new approaches employing both transition-metal-assisted and metal-free synthetic strategies. Examples include the synthesis of substituted quinazolin-4(3*H*)-ones from 3-indolinone-2-carboxylates and nitrosoarenes in the presence of Cs_2_CO_3_ under oxidant- and metal-free conditions [17]; the construction of the quinazolin-4(3*H*)-one core from the reaction of quinazoline-3-oxides with primary amines in the presence of *tert*-butyl hydroperoxide (TBHP), under metal-free reaction conditions [18]; and the preparation of 3-alkyl(aryl)-2-alkyl(aryl)aminoquinazolin-4(3*H*)-ones via (La[N(TMS)_2_]_3_)-catalyzed guanylation/cyclization of ethyl 2-aminobenzoates and symmetrical or unsymmetrical carbodiimides bearing aromatic or/and aliphatic substituents [19].

Multicomponent reactions (MCRs) represent one of the most efficient and versatile approaches for the synthesizing new organic compounds, including biologically active quinazolinone derivatives. They enable the rapid and cost-effective construction of complex structures through the formation of multiple bonds in a single synthetic step, often under mild and environmentally friendly conditions [20]. Moreover, MCRs offer several advantages, such as enhanced efficiency, reduced waste generation and purification steps, and lower solvent usage [21]. MCRs are particularly attractive in the fields of medicinal chemistry due to the ability to rapidly generate diverse libraries of chemical compounds from readily available starting materials [22].

This mini-review highlights selected examples of the multicomponent-reaction-based synthesis of various substituted quinazolin-4(3*H*)-one and 2,3-dihydroquinazolin-4(1*H*)-one derivatives, published between 2011 and 2025. The reported methods were categorized into three main groups:

(1) Metal-catalyzed reactions, employing catalysts, such as palladium, copper, ruthenium, cobalt, and iridium;
(2) Isatoic-anhydride-based strategies, utilizing isatoic anhydride as a key starting material for the construction of quinazolinone ring;
(3) Alternative synthetic approaches involving, among others, the utilization of *N*-(2-aminobenzoyl)benzotriazoles or aryldiazonium salts as efficient building blocks.

## 2. Metal (Palladium, Copper, Ruthenium, Cobalt, Iridium)-Catalyzed Reactions (Scheme 1)

### 2.1. Palladium-Catalyzed Multicomponent Synthesis of Quinazolinones

**Scheme 1:**
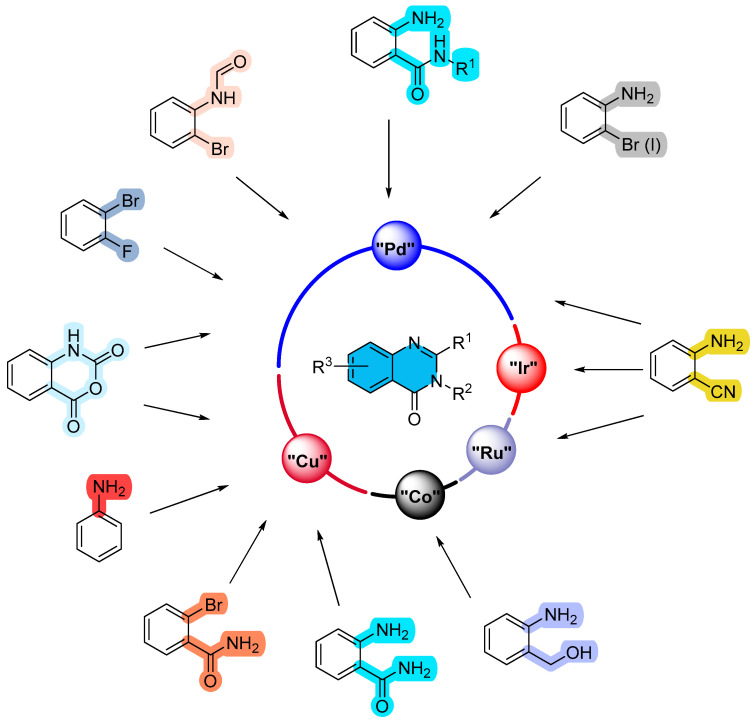
Construction of the quinazolinone scaffold based on metal-catalyzed strategies.

Palladium-catalyzed coupling reactions represent a powerful tool in organic synthesis [23]. They have also found numerous applications in the preparation of various quinazolinone derivatives (Scheme 1). One of the routes to the synthesis of quinazolin-4(3*H*)-one skeletons involves palladium-catalyzed carbonylative transformations of organohalides. In this approach, carbon monoxide serves as an inexpensive carbon source, enabling the efficient preparation of carbonyl-containing compounds by introducing one or more CO molecules.

**Scheme 2:**
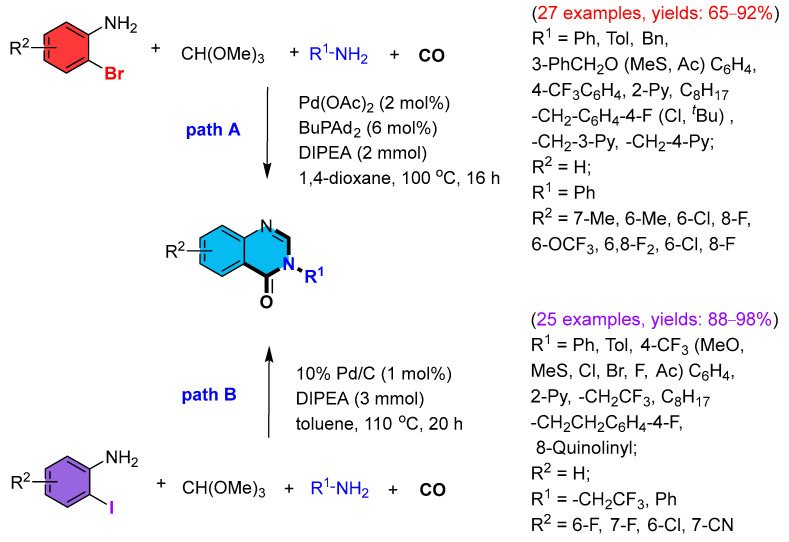
Pd-mediated four-component coupling reaction for synthesis of quinazolin-4(3*H*)-ones from 2-haloanilines: path A [24], path B [25].

This strategy was applied by Wu et al. in 2014 [24]. They developed a palladium-catalyzed four-component reaction for the one-pot synthesis of *N*-aryl(alkyl)-substituted quinazolin-4(3*H*)-ones (Scheme 2, path A). The reaction employed a wide range of commercially available 2-bromoanilines, structurally diverse amines, and ortho esters (trimethyl orthoformate, triethyl orthoformate) along with carbon monoxide (10-bar CO) to provide the desired quinazolinones in good-to-excellent yields (65–92%). The catalytic system consisted of Pd(OAc)_2_ and air-stable di-1-adamantyl-*n*-butylphosphine (BuPAd_2_) with *N*,*N*-diisopropylethylamine (DIPEA) as a base. The reaction’s high selectivity, coupled with its ability to proceed under mild reaction conditions (1,4-dioxane was used as a solvent, 100 °C), improved the economic and environmental impact of this palladium-catalyzed carbonyl coupling process. The Pd(OAc)_2_/BuPAd_2_ catalytic system proved to be highly versatile, tolerating a wide range of substrates—including various substituted 2-bromoanilines and both aliphatic and aromatic amines bearing electron-donating or electron-withdrawing groups under reaction conditions. In addition, this four-component synthesis of quinazolin-4(3*H*)-ones can be easily scaled-up, which is one of its advantages.

**Scheme 3:**
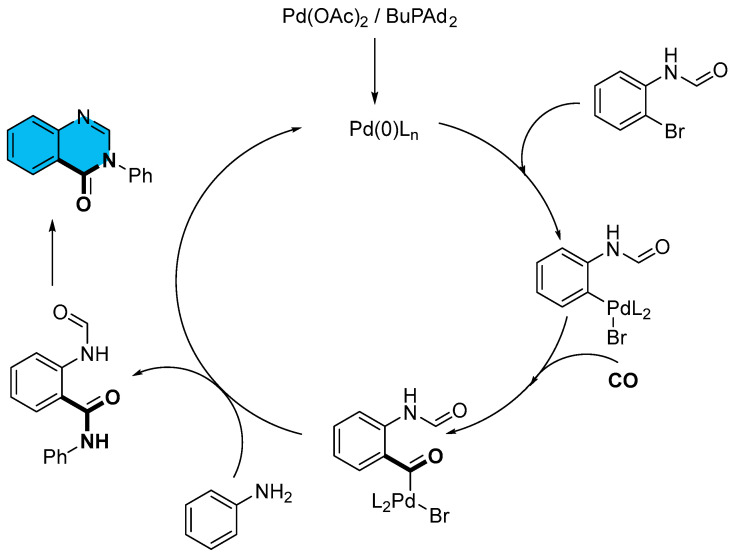
Reaction mechanism for Pd-mediated synthesis of 2-substituted quinazolinones, as suggested by Wu et al. [24].

One of the plausible pathways for the reaction (Scheme 3) begins with the initial reaction of 2-bromoaniline with trimethyl orthoformate, leading to the formation of *N*-(2-bromophenyl)formamide, a key intermediate. Oxidative addition of the Pd(0)L_n_ complex generated in situ to this intermediate, followed by carbon monoxide insertion, yields an acylpalladium complex.

The reaction is completed by the nucleophilic attack of aniline on the acylpalladium complex and subsequent intramolecular condensation of 2-formamido-*N*-phenylbenzamide to afford the target quinazolinone.

**Scheme 4:**
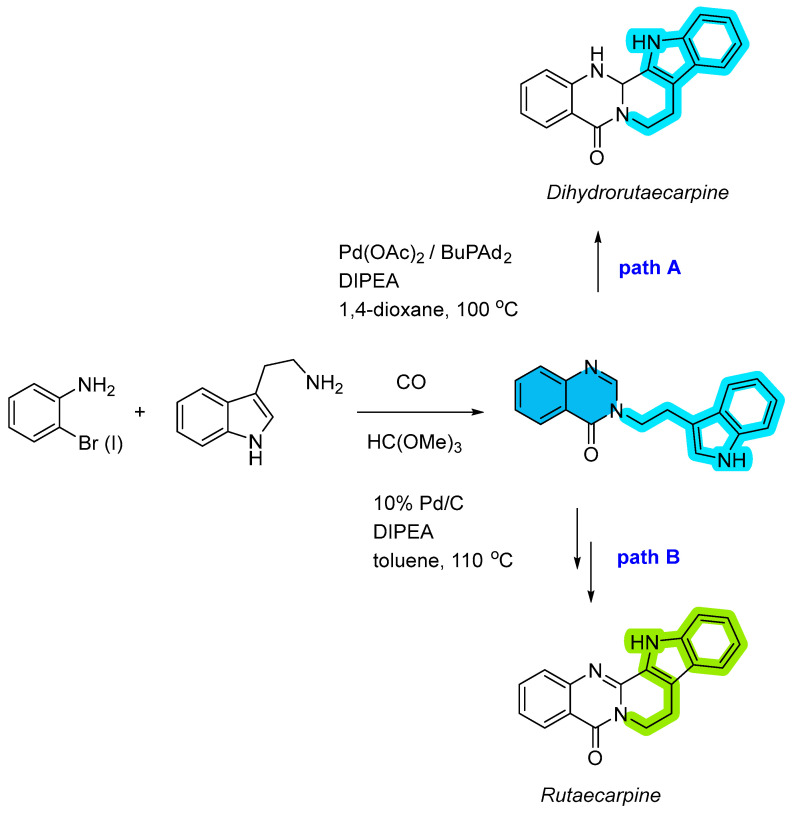
Synthesis of Dihydrorutaecarpine [24] and Rutaecarpine [25].

The approach was successfully applied to the synthesis of a precursor of the bioactive alkaloid Dihydrorutaecarpine, (Scheme 4, path A) demonstrating its potential in medicinal chemistry. However, it is reliant on an expensive and non-recyclable homogeneous catalytic system, which makes it less cost-effective.

In contrast, Pd/C is a readily available, inexpensive, and effective heterogeneous catalyst. In their study, Wu et al. [25] carried out reactions involving a series of variously substituted 2-iodoanilines, trimethyl orthoformate, with structurally different aromatic or aliphatic amines, including trifluoroethylamine, in the presence of DIPEA as a base and 10% Pd/C as the catalytic system in toluene (110 °C). The desired products were obtained in good-to-excellent yields (88–98%) (Scheme 2, path B). Both electron-donating and electron-withdrawing substituents on 2-iodoaniline were well tolerated under the reaction conditions. However, no reaction occurred when 2-bromoaniline was used as a substrate instead of 2-iodoaniline. Additionally, only trace amounts of the desired quinazolinone were obtained in the case of activated 3-amino-4-bromobenzonitrile. Similarly, as earlier, Wu and coworkers confirmed the usefulness of the described procedure in the synthesis of Rutaecarpine (Scheme 4, path B). Interestingly, the authors also demonstrated that Pd/C could be efficiently recycled, and it was also effective in 1 g scale reactions.

**Scheme 5:**
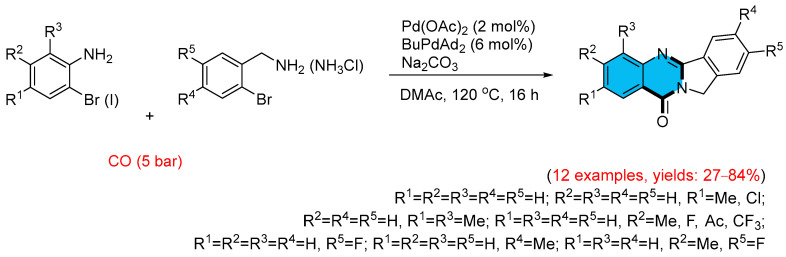
Synthesis of isoindolo[1,2-*b*]quinazolin-10(12*H*)-ones [26].

The same authors, developing a methodology for the synthesis of quinazolin-4(3*H*)-ones, based on the palladium-catalyzed carbonylation [26], described a novel and efficient strategy for construction of tetracyclic quinazolinones, specifically isoindolo[1,2-*b*]quinazolin-10(12*H*)-ones (Scheme 5). Starting from commercially available 2-bromo- or 2-iodo- anilines and 2-bromobenzylamines, the targeted products were obtained in good yields (27–84%) by incorporating two molecules of carbon monoxide. Wu et al. [26] employed a previously used Pd(OAc)_2_/BuPAd_2_ catalyst system under 5 bar of CO in *N*,*N*-dimethylacetamide (DMAc) at 120 °C, with Na_2_CO_3_ (3 eq.) as a base. The reaction showed good tolerance to various substituents on both aniline and benzylamine substrates, including Me, F, Cl, and CF_3_ groups. Importantly, the methodology remained effective even at reduced CO pressure (as low as 2 bars) and also when using stable ammonium salts instead of free benzylamines. However, attempts to use (2-bromophenyl)hydrazine, 2-bromobenzamide, or 2-bromobenzenesulfonamide as analogs of 2-bromobenzylamine did not yield the desired products.

**Scheme 6:**
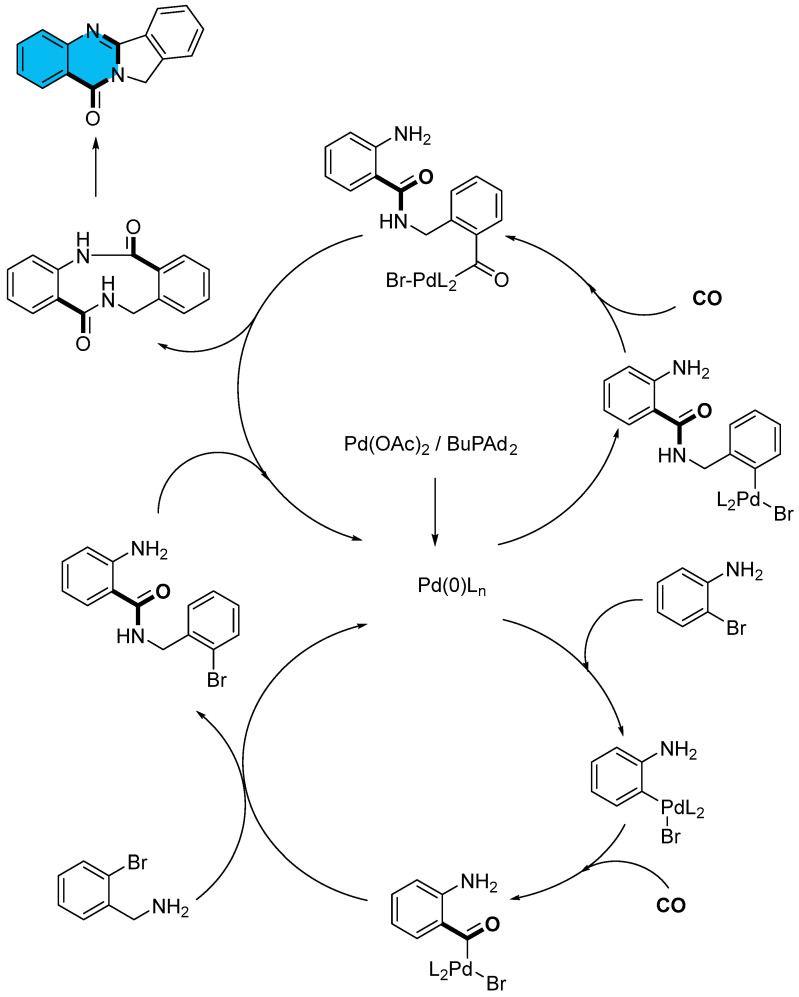
The catalytic cycle proposed for the synthesis of isoindolo[1,2-*b*]quinazolin-10(12*H*)-ones [26].

One of the possible reaction pathways proposed by authors (Scheme 6) involved the oxidative addition of Pd(0) to 2-bromoaniline, followed by insertion of CO and the reaction of the formed acylpalladium complex with 2-bromobenzyl amine providing 2-amino-*N*-(2-bromobenzyl)benzamide, as a stable intermediate produced in situ, which participated in the second catalytic cycle with Pd(0). The key intermediate 11,12-dihydro-5*H*-dibenzo[*b*,*g*][1,5]diazonine-6,13-dione, formed after inserting the second CO molecule, finally led to the product after the attack of *N*-benzylic amide nitrogen atom on the second carbonyl group and the elimination of water.

Other efficient palladium-catalyzed MCRs toward the construction of a quinazolin-4(3*H*)-one core include the use of 2-aminobenzamides [27,28,29,30] and the formation of a C-N bond in the reaction with aryl (heteroaryl) halides via palladium-catalyzed carbon monoxide [27,28] or isocyanide insertion [29]. Alternatively, the quinazolinone skeleton can also be obtained by a palladium-catalyzed three-component coupling of accessible 2-amino-*N*-substituted benzamides with arylboronic acids and isocyanides [30].

**Scheme 7:**
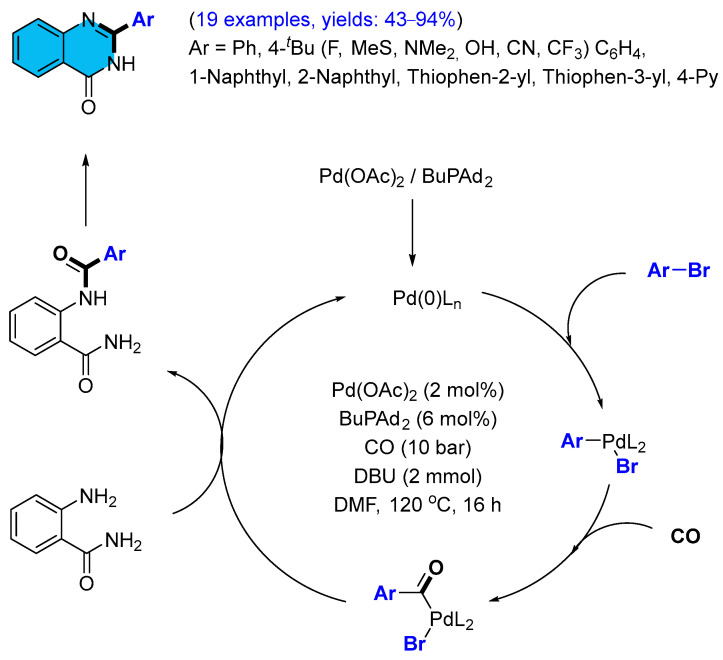
Strategy of the Pd-catalyzed synthesis of 2-arylquinazolinones from anthranilamide, aryl bromide, and CO [27].

Wu and Beller [27] described the synthesis of 2-arylquinazolinones in a palladium-catalyzed carbonylation reaction between anthranilamide and aryl or heteroaryl bromides by using carbon monoxide (CO, 10 bar) as a carbonyl source. The reactions were carried out at 120 °C for 16 h in DMF in the presence of Pd(OAc)_2_ (2 mol%), di-1-adamantyl-n-butylphosphine (BuPAd_2_) (6 mol%), and 1,8-diazabicyclo[5.4.0]undec-7-ene (DBU) as the base (Scheme 7). Various substituted quinazolinones were produced in moderate-to-excellent yields (43–96%). Suddenly, a lower yield of 2-phenylquinazolin-4(3*H*)-one was obtained when iodobenzene was used instead of bromobenzene (30%), and no desired product was formed in the reaction of benzyl chloride under standard conditions. As the authors point out, all products were purified by simple filtration or recrystallization, and chromatography was not needed.

The mechanism proposed for this carbonylative cyclization is displayed in Scheme 7. The reaction began with the oxidative addition of Pd(0) to bromobenzene, followed by the insertion of CO into an arylpalladium complex, giving the key intermediate acylpalladium species. In the next step, following the nucleophilic attack of 2-aminobenzamide on the palladium complex, *N*-(2-carbamoylphenyl)benzamide was formed, which, under intramolecular condensation, led to the formation of quinazolin-4(3*H*)-one. In the authors’ opinion, these steps were responsible for the relatively high reaction temperature required. Notwithstanding, the palladium-catalyzed carbonylative transformation has been shown to be a unique, powerful, and versatile tool for synthesizing quinazolinone skeletons.

Although palladium-catalyzed carbonylation reactions are efficient in the construction of quinazolinones, their potential industrial applications are limited when using homogeneous palladium complexes, such as Pd(OAc)_2_/BuPAd_2_. As mentioned earlier, these systems are often expensive, non-recyclable, and difficult to separate from the product mixture. The solution to these problems, in addition to the already described use of C/Pd, may involve the immobilization of the homogeneous palladium catalysts on porous materials with high surface areas. This approach facilitates separation from the reaction mixture and permits multiple recycling while maintaining the activity of the catalytic center.

**Scheme 8:**
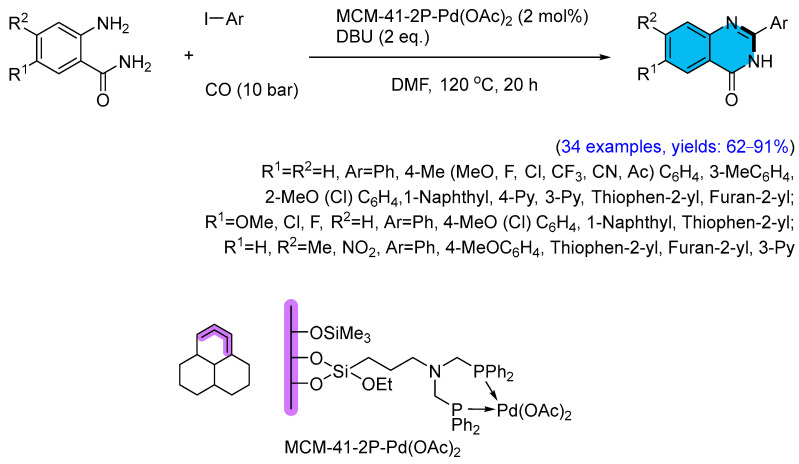
The application of [MCM-41-2P-Pd(OAc)_2_] in the synthesis of 2-arylquinazolinones [28].

Cai et al. [28] investigated the synthesis and application of MCM-41-immobilized bidentate phosphine palladium(II) complex [MCM-41-2P-Pd(OAc)_2_] to carbonylative coupling of 2-aminobenzamides with aryl iodides under CO pressure (10 bar) (Scheme 8). MCM-41 mesoporous materials have proven to be good supports for the immobilization of homogeneous catalysts due to their high surface area, large pore sizes, large pore volumes, and the presence of a large number of silanol groups (Si-OH) on the inner surface. The heterogeneous palladium catalyst obtained by the authors showed high catalytic activity in the reactions. Moreover, it can be easily recovered by a simple filtration process after the reactions and has demonstrated the capability of being recycled up to eight times without significant loss of activity.

The optimized reaction conditions involved the use of MCM-41-2P-Pd(OAc)_2_ (2 mol%) and DBU (2 eq.) as a base in DMF as the solvent at 120 °C under 10 bar of CO for 20 h. Under these conditions, a wide variety of quinazolinone derivatives were obtained in good-to-excellent yields (62–91%, Scheme 8). The reaction also exhibited broad tolerance toward various functional groups present on both the benzene ring of the amide and the aromatic ring of the aryl iodide, regardless of their electronic properties and positions. However, aryl iodides bearing electron-withdrawing groups generally showed lower reactivity compared to those with electron-donating groups. Also, 2-aminobenzamides containing electron-rich substituents demonstrated higher reactivity than the electron-deficient one. Bulky substrates such as 1-iodonaphthalene, as well as heteroaryl iodides including 2-iodofuran, 2-iodothiophene, or 4-iodopyridine, were well-tolerated under the reaction conditions, affording the corresponding 2-(naphthalen-1-yl) and 2-heteroaryl substituted quinazolinones in good yields.

**Scheme 9:**
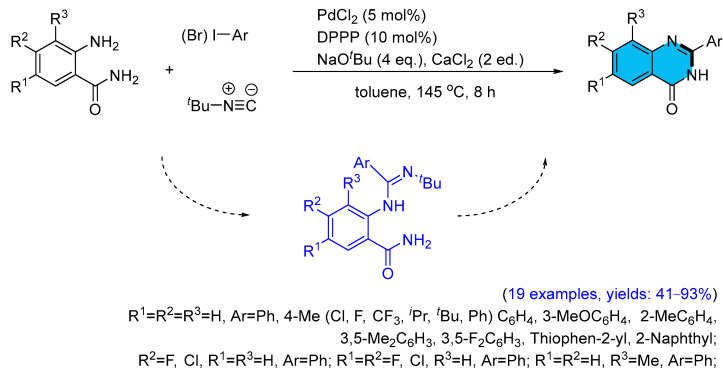
Synthesis of 2-arylquinazolin-4(3*H*)-ones from the reaction of 2-aminobenzamides with aryl halides and *tert*-butyl isocyanide [29].

To eliminate the use of toxic carbon monoxide under high-pressure conditions, Ji and Zhu [29] developed a simple operational method for preparing quinazolin-4(3*H*)-ones from 2-aminobenzamides and aryl halides with isocyanide insertion/cyclization in one step by an efficient palladium-catalyzed three-component reaction (Scheme 9). The best results were obtained using a palladium dichloride/1,3- bis(diphenylphosphino)propane (DPPP) catalyst system, NaO*^t^*Bu as a base, and CaCl_2_ as a drying agent for improved yields. The reactions were carried out in toluene at 145 °C for 8 h under nitrogen. The reaction showed good tolerance to various substituents on aryl iodides and anthranilamides, yielding the target quinazolinones in moderate-to-excellent yields (up to 93%). However, aryl iodides bearing electron-donating groups generally gave higher yields than electron-withdrawing ones. The reaction also worked with some aryl bromides and heteroaryl halides, affording the quinazolinones with good yields.

A plausible mechanism involves the oxidative addition of Pd(0) to the aryl halide, followed by insertion of *tert*-butyl isocyanide, and the addition of 2-aminobenzamide under the assistance of NaO*^t^*Bu, followed by the cyclization of a 2-(*N*’-(*tert*-butyl)arylimidamido)benzamide intermediate (Scheme 9) with the elimination of *tert*-butylamine. The approach, reported by Ji and Zhu [29], required 4 eq. of base, which is not compatible with base-sensitive functional groups in the substrates. Furthermore, this route is not suitable for synthesizing 2,3-disubstituted quinazolinones.

**Scheme 10:**
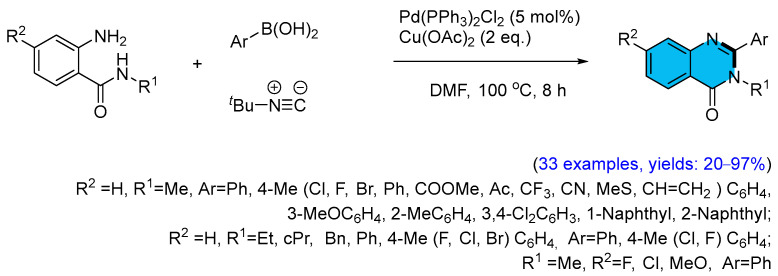
The preparation of 2,3-disubstituted quinazolinones in the reaction of anthranilamides with isocyanide and arylboronic acids [30].

With this in mind, Zhu and Yang [30] proposed a new approach for the preparation of 2,3-disubstituted quinazolinones involving the palladium-catalyzed oxidative coupling of anthranilamides with isocyanides and arylboronic acids (Scheme 10). The optimized reaction conditions included the use of Pd(PPh_3_)_2_Cl_2_ (5 mol%) as the catalyst and Cu(OAc)_2_ (2 eq.) as the oxidant and carrying out the reactions under a nitrogen atmosphere at 100 °C for 8 h in DMF in a sealed tube. Interestingly, when the reaction was performed under free air or oxygen atmospheres without Cu(OAc)_2_, no desired product was detected. A wide variety of arylboronic acids bearing either electron-withdrawing or electron-donating substituents on the aryl ring were successfully transformed into the corresponding products. Similarly, most 2-amino-*N*-alkyl- and *N*-aryl-benzamides, including those substituted on the benzene ring, produced the targeted quinazolinones in moderate-to-good yields. To the authors’ surprise, the use of 2-aminobenzamide as a substrate did not result in the desired product.

From a synthetic point of view, methodologies based on the widely available substrates as starting materials are more attractive and necessary as synthetic tools. A potential issue with the strategies outlined above is the availability of suitably substituted 2-aminobenzamides. On the other hand, 2-aminobenzonitrile derivatives are broadly available and could be used as precursors to the aforementioned anthranilamides, thereby improving substrate versatility.

**Scheme 11:**
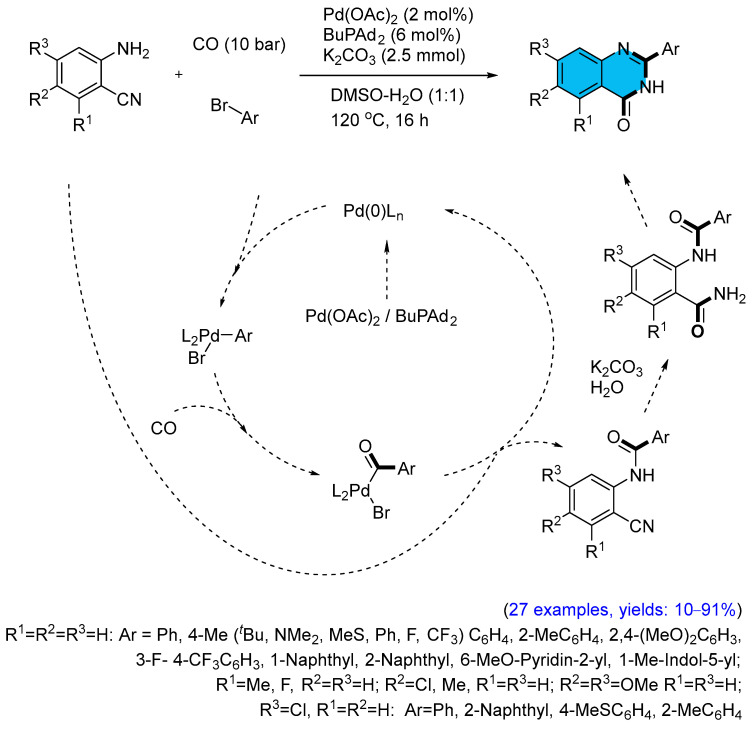
Synthesis of 2-arylquinazolinones from 2-aminobenzonitriles and bromobenzenes via Pd(OAc)_2_/BuPAd_2_-catalyzed carbonylation [31].

Building on these premises, Beller and Wu [31] developed a simple carbonylative procedure for the synthesis of quinazolinones from readily available 2-aminobenzonitriles and bromobenzenes via Pd(OAc)_2_/BuPAd_2_-catalyzed carbonylation (CO 10 bar) in a DMSO-H_2_O (1:1) solution in the presence of inexpensive K_2_CO_3_ as a base (Scheme 11). Based on the established procedure, a series of differently substituted quinazolinones were obtained in yields of 10–91% under identical reaction conditions. Importantly, all the products were isolated only by recrystallization. It turned out that bromoarenes with typical electron-donating functional groups, such as methyl-, methoxy-, *tert*-butyl-, or *N*,*N*-dimethylamino, produced the corresponding quinazolinones in 74–91% yields. On the other hand, aryl bromides bearing electron-withdrawing substituents led to required lactams in slightly lower but still good yields (61–73%). Remarkably, heteroaryl bromides were also successfully applied in this transformation, delivering the desired products with a similar yield of 61–71%. In the case of 2-aminobenzonitrile components, methyl-, methoxy-, fluoro-, and chloro- were all well-tolerated, and the corresponding 2-(phenyl, 2-methylphenyl, 4-methylsulfanylophenyl) quinazolinones were obtained in good yields of 55–87%. An exception was the 5-nitro derivative, which yielded only 10% of the desired product in the reaction with bromobenzene, presumably due to side reactions involving the nitro group.

It can be surmised that the reaction proceeded via the aminocarbonylation of aryl bromides in a catalytic cycle, resulting in the formation of *N*-(2-cyanophenyl)benzamide. Further hydrolysis with K_2_CO_3_ produced *N*-(2-carbamoylphenyl)benzamide, which then underwent intramolecular condensation to yield the quinazolinone product (lower part of Scheme 11).

**Scheme 12:**
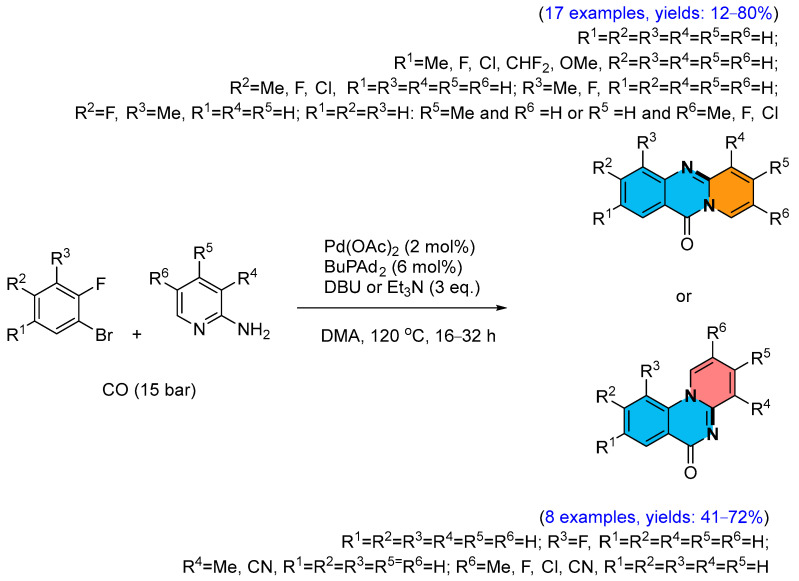
Synthesis of linear and angular fused pyridoquinazolones [32].

Condensed pyridoquinazolones are important due to their broad biological activity, including antimicrobial, anti-inflammatory, and CNS-related activities. Based on their experience in the synthesis of quinozalinones via palladium-catalyzed reactions, Wu and Beller [32] presented a novel and efficient strategy for synthesizing linear or angular fused pyridoquinazolone derivatives (Scheme 12). The significance of this methodology lies in the base-controlled selectivity, which enables the preparation of either linear or angular isomers by simply changing the base used in the reaction. DBU (1,8-diazabicyclo[5.4.0]undec-7-ene), a stronger organic base (pKa = 12), promoted the formation of linear quinazolinone products, whereas using triethylamine (Et_3_N, pKa = 10.8) led to the reaction forming angular isomers. NMR spectroscopic studies showed that DBU can deprotonate 2-aminopyridine and to generate the 2-imino-2*H*-pyridin-1-ide, which was not observed in the presence of Et_3_N.

Therefore, the deprotonated form of 2-aminopyridine underwent nucleophilic attack on the acyl-palladium intermediate, ultimately yielding linear products, after intramolecular nucleophilic aromatic substitution. In contrast, the use of Et_3_N led to a different pathway, resulting in angular fused products. The methodology described by Wu demonstrates good tolerance for a wide range of substrates, bearing both electron-withdrawing and electron-donating groups on 1-bromo-2-fluorobenzene (e.g., F, Cl, Ac, Me) as well as on the 2-aminopyridine ring (e.g., Me, Cl, F). However, during the synthesis of linear fused pyridoquinazolones using 2-amino-5-fluoro (5-chloro) pyridines, corresponding dehalogenated products were also isolated, either as a mixture with a related halogenoderivative (for fluorine) or as the sole product (for chlorine). Furthermore, more sterically hindered substrates afforded only the corresponding amides and did not form pyridoquinazolones. In contrast, in the case of the construction of the angular products, steric and electronic properties of the substrates did not influence the reaction outcome.

**Scheme 13:**
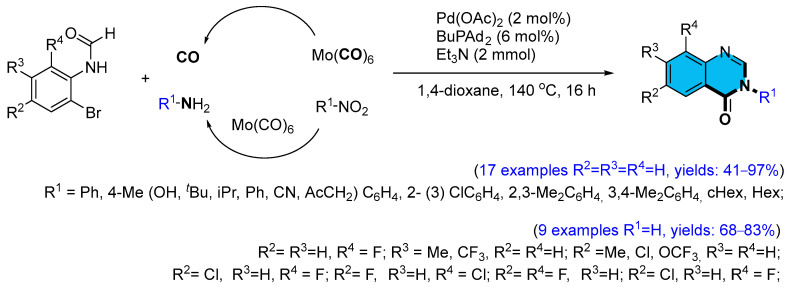
Mo(CO)_6_ as a multifunctional reactant in the synthesis of 2-substituted quinazolinones [33].

Nevertheless, the difficulty of using gaseous CO in experiments and its toxicity limit the usefulness of this and other strategies based on the direct use of carbon monoxide. Wu et al. [33] reported the carbonylation of 2-bromoformanilides (Scheme 13) with the generation of in situ aromatic or aliphatic amines from corresponding nitro compounds. The process employed Mo(CO)_6_ as a multifunctional reactant, acting both as a CO-releasing reagent and as a reductant. Molybdenum hexacarbonyl proved to be the most effective source of CO among the tested carbonyl metal complexes, including Co_2_(CO)_8_ or Fe_3_(CO)_12_, which additionally promoted the reduction in nitro compounds. In contrast, when carbon monoxide was used instead of metal complexes, a low conversion of both starting nitrobenzene and 2-bromoformanilide was observed. The reactions were carried out in 1,4-dioxane at 140 °C using Pd(OAc)_2_ (2 mol%) and di-1-adamantyl-*n*-butylphosphine (BuPAd_2_) (6 mol%) as the optimal catalytic system and Et_3_N (2 mmol) as the base. In the absence of a phosphine ligand or the presence of other monodentate and bidentate ligands, such as PPh_3_, PCy_3_, Xantphos, DPPB, BINAP, and DPPF, the yields were significantly lower. The desired quinazolin-4(3*H*)-ones bearing various substituents at the lactam nitrogen atom and at positions 6, 7, or 8 of the benzene ring were obtained in moderate-to-excellent yields (41–97%), indicating that the reaction conditions were compatible with a wide range of substrates.

**Scheme 14:**
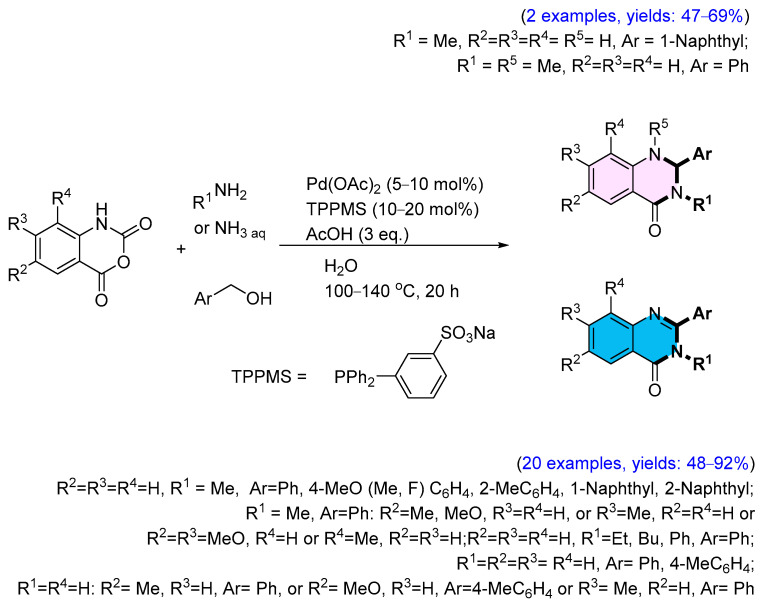
Synthesis of 2- and 2,3-disubstituted quinazolinones from benzylic alcohols, isatoic anhydrides, and amines or ammonia [34].

In 2021, Hikawa et al. [34] presented a novel and environmentally friendly method for synthesizing 2-aryl quinazolinones directly from benzylic alcohols, avoiding the use of unstable aldehydes, isatoic anhydrides, and amines or ammonia (Scheme 14). The reaction was carried out in water as a solvent and catalyzed by the Pd(OAc)_2_/sodium diphenylphosphinobenzene-3-sulfonate (TPPMS) system. The studies on the optimization of the model reaction conditions (benzyl alcohol, isatoic anhydride, and MeNH_2_—40% aqueous solution) clearly showed that the use of MeNH_2_·AcOH (in an equimolar ratio—3 mmol), instead of methylamine alone, significantly improved the yields of both cyclized products formed in this reaction: 3-methyl-2-phenylquinazolin-4(3*H*)-one (from 3% to 29%) and 3-methyl-2-phenyl-2,3-dihydroquinazolin-4(1*H*)-one (from 15% to 38%). Moreover, the use of MeNH_2_ (2.5 mmol) and AcOH (3 mmol) proved critical for directing the reaction outcome toward the formation of 3-methyl-2-phenylquinazolin-4(3*H*)-one as the main product, which was obtained in 90% yield. The use of water as the solvent was similarly essential. Attempts to replace water with other solvents (e.g., toluene, DMSO, or EtOH) proved ineffective, as did efforts to use alternative catalytic systems, such as PdCl_2_, Pd(TFA)_2_, or Pd_2_(dba)_3_ CHCl_3_ in combination with TPPMS. Isatoic anhydrides substituted with electron-donating groups (OMe and Me), an electron-withdrawing group (Cl), or bearing sterically demanding substituents were well-tolerated under reaction conditions, affording the desired products in excellent yields (68–90%). Similarly, benzylic alcohols bearing electron-donating groups (e.g., Me, MeO) or an electron-withdrawing fluoro atom in the *para* position proved to be effective components in MCR. However, no product formation was observed when 4-nitrobenzyl alcohol or 2-phenylethyl alcohol were employed, likely due to the failure to generate the corresponding π-benzyl Pd(II) cation species. In general, both aliphatic and aromatic amines, as well as ammonia, demonstrated good reactivity as nitrogen sources, enabling the effective construction of 2,3-disubstituted quinazolinones.

**Scheme 15:**
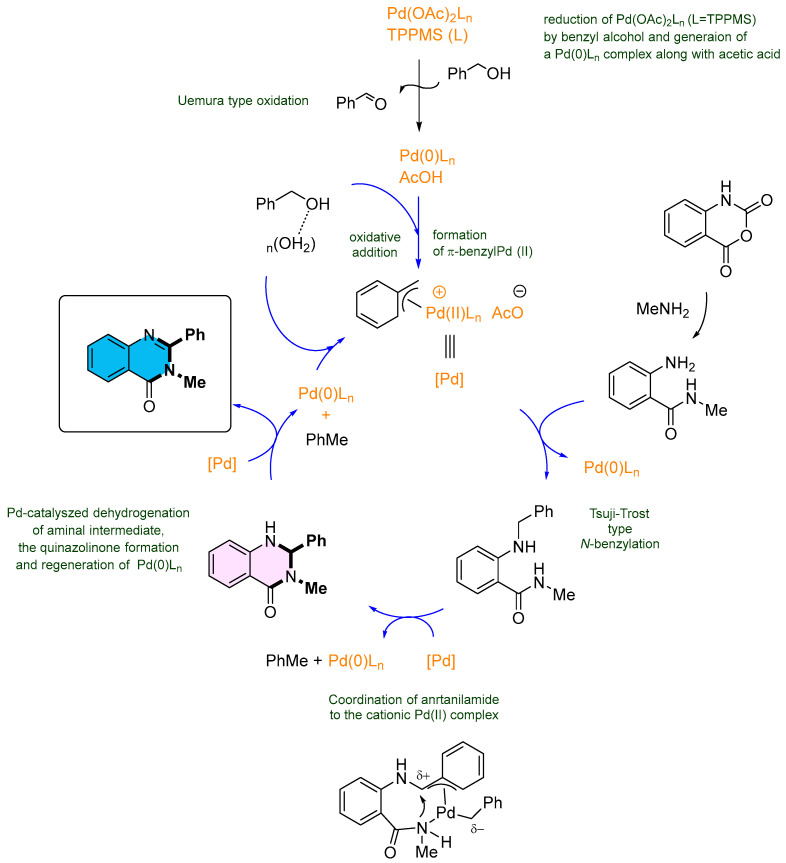
Proposed mechanism for the Pd-catalyzed reaction of benzylic alcohols with isatoic anhydrides and amines or ammonia [34].

The presented strategy represented an extension of previous research in the field of quinazolinone synthesis and was based on the domino reaction sequence involving *N*-benzylation reaction, benzylic C−H amidation, and dehydrogenation [35]. The plausible pathway for the five-step Hikawa synthesis of 2- or 2,3-disubstituted quinazolinones is illustrated in Scheme 15. The first stage involved generating the key intermediate: π-benzylPd(II) cation. This was achieved by the reaction of Pd(OAc)_2_L_n_ (L = TPPMS) with benzyl alcohol initially leading to the formation of the active Pd(0)L_n_ complex (along with AcOH and a corresponding benzaldehyde via Uemura-type oxidation in water), which was then converted to the π-benzylPd(II) cation through oxidative addition of an alcohol [36]. Two main factors contributed to the success of this stage: the use of water as a solvent and the addition of acetic acid. On the one hand, the hydroxy groups of water activated the carbon–oxygen bond of benzyl alcohol through the formation of a hydrogen bond network, thereby facilitating the formation of the Pd(II) complex. On the other hand, water molecules helped stabilize this complex. Moreover, the presence of acetic acid accelerated the formation of the π-benzylPd(II) species. The next stages included the in situ generation of anthranilamide derivative from isatoic anhydride and amine, followed by its attack on the cationic π-benzylpalladium(II) intermediate via dehydrative Tsuji–Trost-type *N*-benzylation to yield the *N*-benzylated anthranilamide. This was followed by amide-directed benzylic C–H amination via a π-benzylpalladium(II) system, generating the dihydroquinazolinone intermediate. Finally, Pd-catalyzed dehydrogenation of the dihydroquinazolinone intermediate afforded the quinazolinone product.

### 2.2. Copper-Catalyzed Multicomponent Synthesis of Quinazolinones

Copper salts have emerged as a promising alternative to more expensive catalytic systems, such as those based on palladium. The effectiveness of copper-based catalysis is well documented in the literature, particularly in cross-coupling reactions for the synthesis of numerous bioactive molecules and natural products. This is largely due to their good tolerance of various functional groups, low toxicity, and cost-effectiveness [37,38].

Recognizing these features, Guo et al. [39] developed a one-pot procedure for the synthesis of 2-substituted and 2,3-disubstituted quinazolinones using a three-component, copper-catalyzed tandem reaction of 2-bromobenzamide or *N*-alkyl(aryl)-2-bromobenzamide derivatives with aldehydes and aqueous ammonia, offering a more practical and efficient route to the target molecules.

**Scheme 16:**
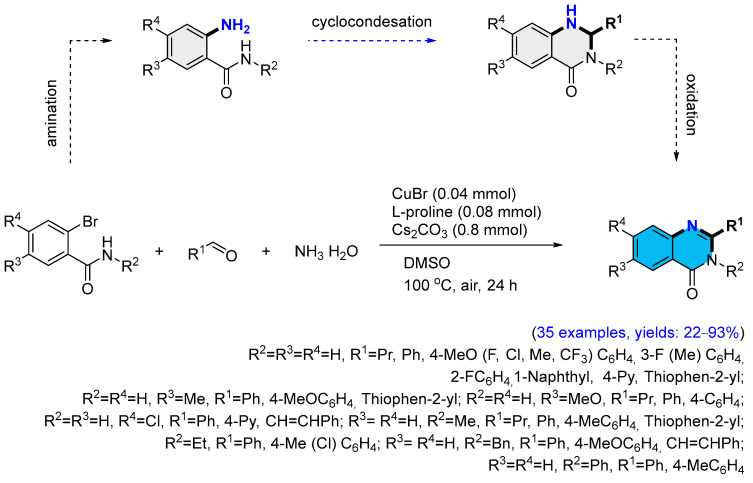
Cu-catalyzed synthesis of 2-substituted and 2,3-disubstituted quinazolinones from 2-bromobenzamide, aldehydes, and aqueous ammonia [39].

**Scheme 17:**
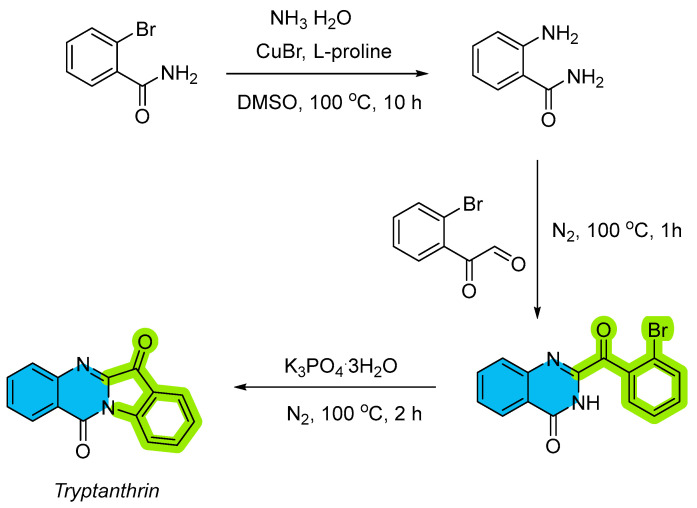
Copper-catalyzed one-pot synthesis of alkaloid Tryptanthrin [39].

An investigation into the scope and limitations of the reaction [39] showed that aryl-substituted aldehydes with either electron-donating or electron-withdrawing groups on the aromatic ring (including Me, MeO, CF_3_, Cl, and F) reacted efficiently, affording the desired products in yields of 67–83%. Similarly, heteroaryl aldehydes also underwent the reaction smoothly. Moreover, it was found that the steric hindrance of aromatic aldehydes had an insignificant impact on the reaction outcome. Furthermore, both alkyl- and alkenyl-substituted aldehydes were well tolerated under the reaction conditions, delivering the desired products in moderate yields. In the synthesis of 2,3-disubstituted quinazolinones, it was found that *N*-alkyl amides (Me, Et, Bn) were more effective than *N*-aryl amides (Ph), yielding the target products in higher yields when reacted with various substituted aldehydes bearing aryl, heteroaryl, alkyl, or alkenyl groups. A plausible mechanism (upper part of Scheme 16) for the formation of 2-substituted and 2,3-disubstituted quinazolinones involves the copper-catalyzed amination of 2-bromoamide with aqueous ammonia in the presence of L-proline as a ligand, followed by cyclocondensation of the 2-aminobenzamide intermediate formed in situ with an aldehyde, and finally, the oxidation of 2,3-dihydroquinazolinone by air in the presence of CuBr to yield the final product. The usefulness of the described methodology was confirmed by the authors in the synthesis of alkaloid Tryptanthrin, which is found in several plants and exhibits potent cytotoxicity against human cell lines (MCF-7, NCI-H460, SF-268) (Scheme 17).

**Scheme 18:**
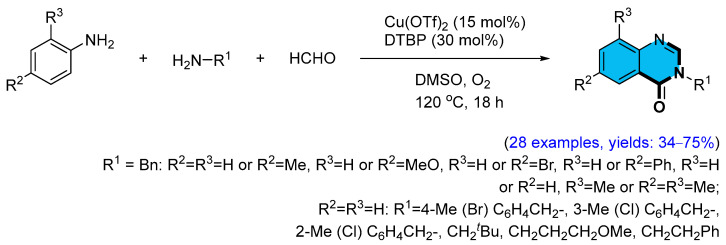
Synthesis of 3-substituted quinazolinones from the reaction of substituted anilines, primary alkyl amines, and HCHO in the presence of Cu(OTf)_2_ [40].

Liang et al. [40] report an efficient method for the synthesis of 3-substituted quinazolinones (Scheme 18) from the reaction of easily accessible substrates, such as various substituted anilines, primary alkyl amines, and formaldehyde (HCHO) as a carbon source, by using Cu(OTf)_2_ and DMSO as catalyst and solvent in the presence of di-*tert*-butyl peroxide (DTBP) and oxygen. The optimized reaction conditions were well tolerated with various functional groups on the aryl ring of anilines (Me, OMe, Br, Cl). Anilines containing electron-rich groups afforded the products in higher yields than those with an electron-withdrawing group, probably because the electron-rich anilines enhance the nucleophilicity of the reaction intermediates and favor the cyclization process. In the case of anilines containing a strong electron-withdrawing group (i.e., CF_3_, CO_2_Et, and NO_2_), the desired product was not formed.

**Scheme 19:**
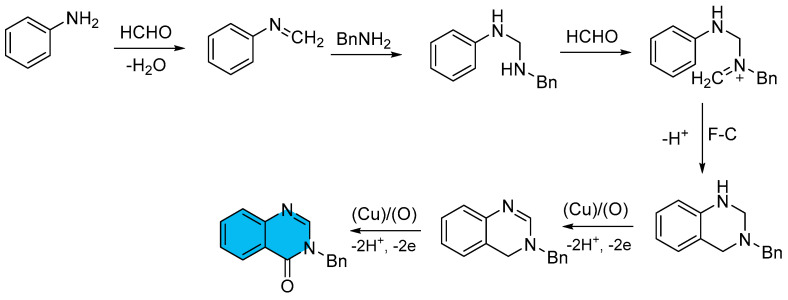
Suggested mechanism for the Cu-catalyzed synthesis of 2-substituted quinazolinones from anilines, primary alkyl amines, and HCHO [40].

As reported by the authors, a small amount of 3-arylquinazolinones was detected when anilines and alkyl amines were used as coupling partners. However, 3-alkylquinazolinones were obtained with high selectivity each time, which can be attributed to the higher reactivity of alkyl amines. Mechanistic studies (Scheme 19) suggest that the reaction likely begins with the formation of an imine from aniline and formaldehyde, followed by the addition of an alkyl amine and subsequent condensation with HCHO to yield an iminium ion.

The next step involves an intramolecular Friedel–Crafts (F-C) aminoalkylation at the *ortho*-position of the aromatic ring, leading to the formation of a cyclic aminal. This intermediate then undergoes single-electron oxidation-induced dehydrogenation under oxidative copper catalysis to form a dihydroquinazoline, which is subsequently converted into the desired product via copper-catalyzed benzylic oxidation. Thus, the entire process involves the formation of three C−N and one C−C bonds in connection with the benzylic functionalization.

**Scheme 20:**
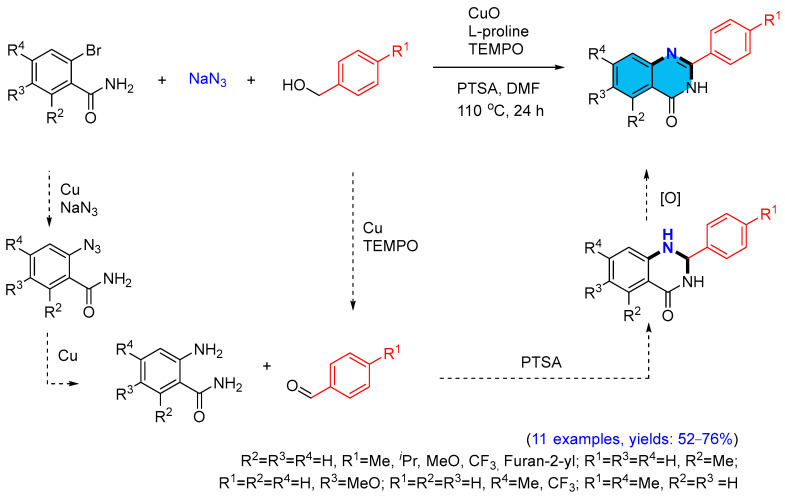
Cu-catalyzed synthesis of 2-arylquinazolinones from 2-bromobenzamides, benzylic alcohols and sodium azide [41].

Shinde and Kshirsagar [41] developed a simple and proficient protocol for the synthesis of 2-arylquinazolinones (Scheme 20). The molecular framework was obtained via a three-component cascade redox reaction between substituted 2-bromobenzamides, benzylic alcohols, and sodium azide as a nitrogen source. The optimal reaction conditions required a CuO/L-proline catalytic system, TEMPO (2,2,6,6-tetramethyl-1-piperidinyloxy) as the oxidant, and *para*-toluenesulfonic acid (PTSA) as an additive in DMF as the solvent. Attempts made by the authors to modify reaction conditions (the nature of the catalyst (e.g., CuI, Cu_2_O, CuCl_2_), ligand (2,2-bipyridine), solvent (DMSO, toluene, DCE), or the nitrogen source (trimethylsilyl azide—TMSN_3_) resulted either in decreased reaction yields or in no product formation. Various functionalized 2-substituted quinazolinone derivatives were synthesized using this protocol. Multicomponent reactions provided good yields (52–76%) and exhibited a broad substrate range for both bromoamides (Me, OMe, CF_3_) and benzyl alcohols (Me, *^i^*Pr, CF_3_).

Based on a series of experiments, the authors [41] proposed a possible reaction pathway that involves, firstly, a copper-catalyzed S_N_Ar reaction of 2-bromobenzamide with NaN_3_, followed by a copper-assisted elimination of N_2_, finally yielding the corresponding amine. In parallel, benzyl alcohol is oxidized in the presence of copper and TEMPO to generate benzaldehyde. In the end, the benzaldehyde formed in situ undergoes oxidative condensation with 2-aminobenzamide to afford the corresponding quinazolinone (lower part of Scheme 20).

**Scheme 21:**
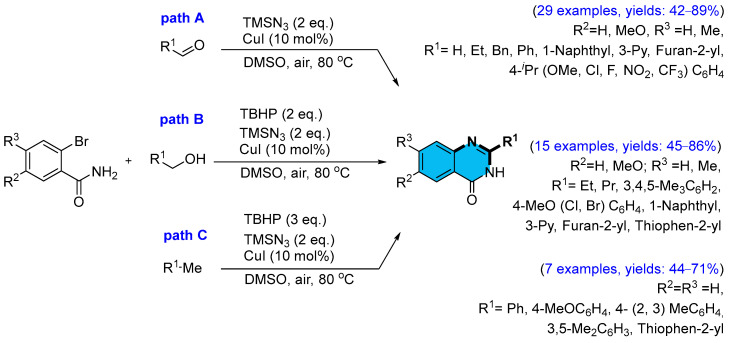
Cu-catalyzed synthesis of 2-substituted quinazolinones through the reactions of 2-bromobenzamides with aldehydes, primary alcohols, or methyl arenes in the presence of TMSN_3_ [42].

Tripathi et al. [42] presented an efficient and cost-effective copper(I)-catalyzed method for synthesizing 2-substituted quinazolinones under ligand- and base-free conditions (Scheme 21). This approach differs from those previously described. The protocol utilized readily available starting materials, including various carbon sources, such as aromatic, aliphatic, and heteroaromatic aldehydes, primary alcohols, or methyl arenes, as coupling partners with 2-bromobenzamides in the presence of trimethylsilyl azide (TMSN_3_) as a nitrogen source. The reaction proceeded via a tandem oxidative process catalyzed by CuI (10 mol%) in DMSO at 80 °C under air or with *tert*-butyl hydroperoxide (TBHP) as the oxidant.

**Scheme 22:**
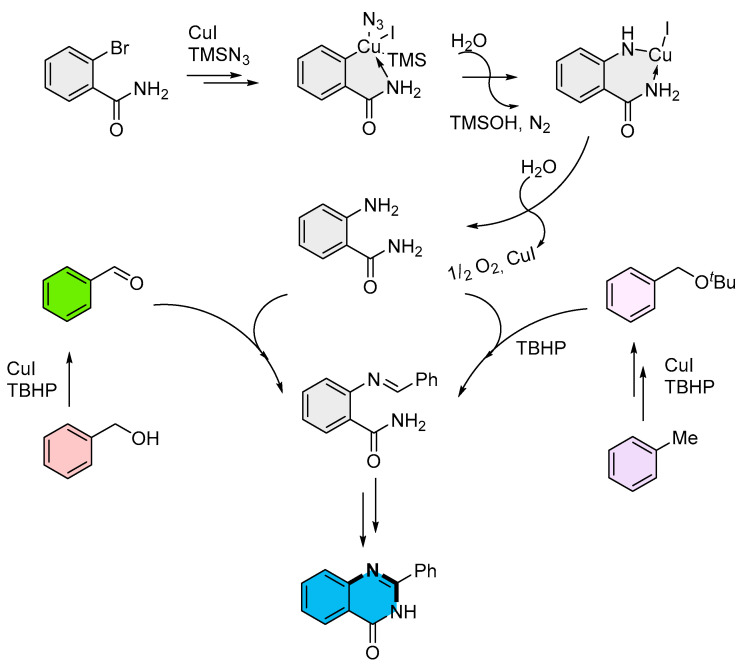
Suggested mechanism by Tripathi et al. for the synthesis of 2-substituted quinazolinones [42].

When aldehydes and alcohols were used, both electron-donating and electron-withdrawing substituents were well tolerated, and the corresponding quinazolinone products were obtained in moderate-to-excellent yields (42–89%). Remarkably, alkyl alcohols also reacted well under the optimized reaction conditions to provide corresponding lactams. However, when alcohols were used, the addition of 2 eq. of TBHP was necessary to oxidize them into the corresponding aldehyde, which served as key intermediates in the synthesis of quinazolinones from alcohols (Scheme 22). Moreover, the authors’ studies indicated the radical nature of this transformation.

Even more TBHP was required when methyl arenes were used as a substrate for the synthesis of quinazolinone; in this case, as much as 3 eq. was needed. Considering this fact and the results of a series of control experiments, the authors suggest that *tert*-butyl benzyl ether is produced during the reaction by coupling benzyl and *tert*-butoxy radicals, which then undergo intermolecular amination by reacting with 2-aminobenzamide, yielding a benzylamine intermediate (Scheme 22). This intermediate is subsequently oxidized to an imine, which further cyclizes to yield the target quinazolinone. In turn, 2-aminobenzamide is produced from 2-bromobenzamide in the catalytic cycle by treating with CuI, TMSN_3_, and water from DMSO. The highest yield was observed for toluene (71%). In contrast, substrates containing electron-donor groups, such as 4-methylanisole or *ortho*-, *meta*-, and *para*-xylenes, produced lower yields (59–67%).

**Scheme 23:**
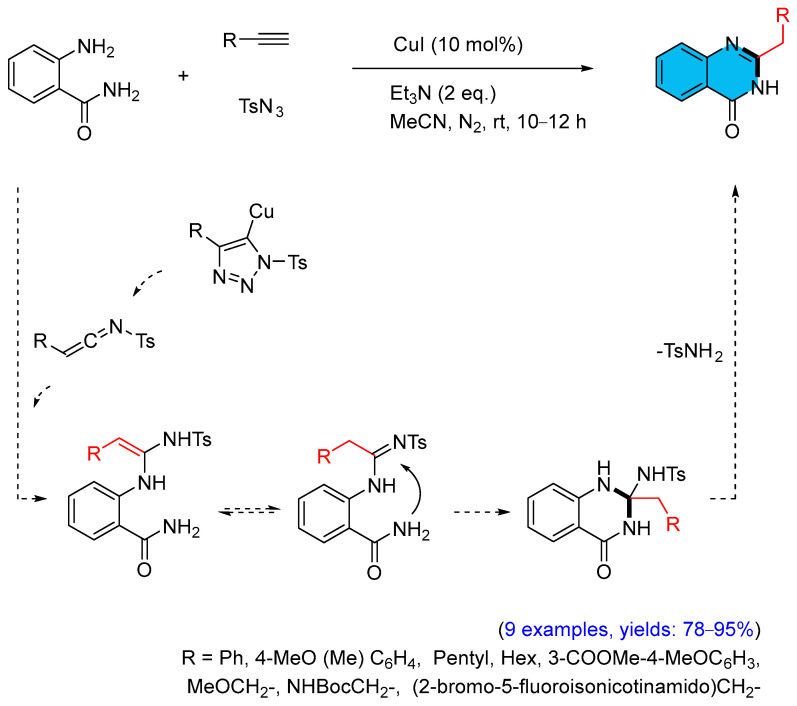
Cu-catalyzed synthesis of 2-substituted quinazolinones via the reaction of 2-aminobenzamide with *p*-toluenesulfonyl azide and terminal alkynes [43].

In 2017, Mulakayala [43] reported a simple, scalable, and straightforward copper-catalyzed strategy for synthesizing 2-substituted quinazolin-4(3*H*)-ones (Scheme 23) via a one-pot three-component reaction of 2-aminobenzamide, *p*-toluenesulfonyl azide (TsN_3_), and terminal alkyne carried out under mild conditions. The optimized reaction conditions—using CuI as the catalyst, Et_3_N as the base, and performing the reaction in MeCN at ambient temperature for 10–12 h—afforded the corresponding products in excellent yields (up to 95%). A broad range of acetylenes, containing aromatic, heteroaromatic, or aliphatic substituents, as well as a carbamate functional group, were well-tolerated in the reaction, leading to the desired quinazolinone. Interestingly, this method was also employed to synthesize 5-substituted pyrazolo[4,3-*d*]pyrimidin-7(6*H*)-ones which can serve as important cores in medicinal chemistry.

The authors’ proposed route for constructing the quinazolinone skeleton is presented in the lower part of Scheme 23. Based on reports from the literature, it can be assumed that the copper acetylide, initially formed from alkyne and CuI, underwent a 1,3-dipolar cycloaddition with *p*-toluenesulfonyl azide, yielding a triazole derivative, which in the subsequent step was transformed into a highly reactive key intermediate—ketenimide. The amine group of antranilamide then attacked ketenimide to form a 2-((1-((4-methylphenyl)sulfonamido)vinyl)amino) benzamide intermediate, which, upon intramolecular cyclization and elimination of TsNH_2_, yielded the final product.

**Scheme 24:**
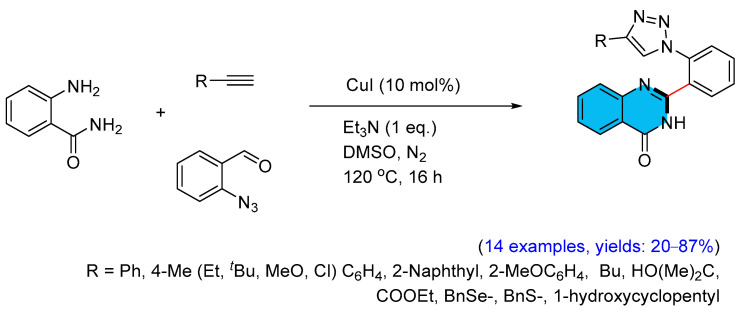
Synthesis of 2-(2-(1,2,3-triazol-1-yl)phenyl)quinazolinones [44].

In turn, Alves et al. [44] developed an efficient and selective protocol for the synthesis of 2-(2-(1,2,3-triazol-1-yl)phenyl)quinazolin-4(3*H*)-one via a copper-catalyzed multicomponent reaction (Scheme 24). Hybrid molecules containing the quinazolinone and 1,2,3-triazole moieties lead to the construction of new compounds with promising pharmacological activities, e.g., antihistaminic activity [45], anticancer activity [46], or for the treatment of Alzheimer’s disease [47].

Similarly to the previously reported method, the process employed anthranilamide and terminal alkynes as substrates, along with 2-azidobenzaldehyde. The reactions were conducted using CuI as the most effective catalyst among the copper salts tested, triethylamine (Et_3_N) as the base, and DMSO as the optimal solvent. Attempts to replace CuI with other copper salts (e.g., CuBr, CuCl, or CuBr_2_) or to use alternative solvents (e.g., toluene, 1,4-dioxane) resulted in significantly reduced yields or failure of the reaction.

As reported by the authors [44], the selectivity of the reaction can be controlled, and depending on the reaction temperature and the amount of Et_3_N used, either 2-(2-(1,2,3-triazol-1-yl)phenyl)quinazolin-4(3*H*)-one or its dihydro analogue can be obtained as the main products. Specifically, conducting the reaction at 100 °C with 2 equivalents of Et_3_N led to the formation of the dihydroquinazolinone derivative in an 82% yield. In contrast, performing the reaction at 120 °C with only 1 equivalent of Et_3_N resulted in the formation of quinazolinone derivatives in moderate-to-good yields (20–87%). The presented protocol exhibited good tolerance toward various substituents on the alkyne moiety. In the case of phenylacetylenes, both electron-donating and electron-withdrawing substituents in the *para* position afforded the expected products in similar yields (54–66%). A decrease in yield was observed for (2-methoxyphenyl)acetylene (2-methylfenyl)acetylene (42%), probably due to increased steric hindrance compared to its *para*-substituted analogue (66%). Other functional groups also exhibited good tolerance on the alkyne, as demonstrated by successful reactions with propargyl alcohols, alkynyl selenides, and alkynyl sulfides.

The reaction proceeded in two main stages. Firstly, the triazole nucleus was formed through the reaction between the terminal alkyne and CuI in the presence of Et_3_N, generating the copper acetylide intermediate. This stage is similar to the process described above in Scheme 23. Subsequently, 2-azidobenzaldehyde reacted with the copper acetylide, finally leading to the formation of the 2-(1,2,3-triazol-1-yl)benzaldehyde intermediate and the regeneration of the copper catalyst within the catalytic cycle. The second stage involved a condensation reaction between the amino group of anthranilamide and the carbonyl group of the 1,2,3-triazol-1-yl derivative of benzaldehyde intermediate, affording the corresponding imine. Next, the intramolecular cyclization of the resulting 2-(benzylideneamino)benzamide occurred, forming the 2,3-dihydroquinazolinone, which was subsequently oxidized in the reaction medium to yield quinazolin-4(3*H*)-one.

**Scheme 25:**
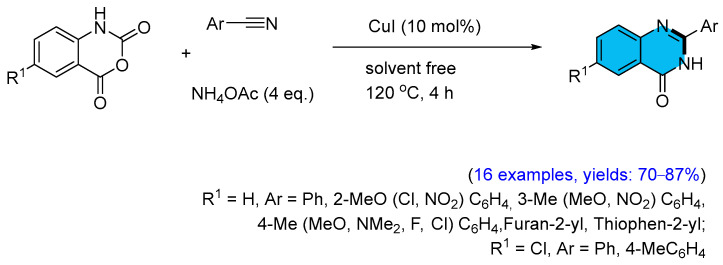
Synthesis of 2-arylquinazolinones [48].

Soheilizad et al. [48] proposed a straightforward protocol for synthesizing quinazolin-4(3*H*)-ones containing an aryl or heteroaryl substituent at the 2 position, using a one-pot, copper(I)-iodide-catalyzed, three-component reaction between isatoic anhydrides, aryl nitriles, and ammonium acetate as an ammonia source (Scheme 25). The optimal reaction conditions involved the use of 4 eq. of NH_4_OAc and 10 mol% CuI at 120 °C for 4 h, under solvent-free conditions, making the process environmentally friendly. In contrast, the use of solvents such as water, DMSO, or toluene was less effective or completely ineffective.

**Scheme 26:**
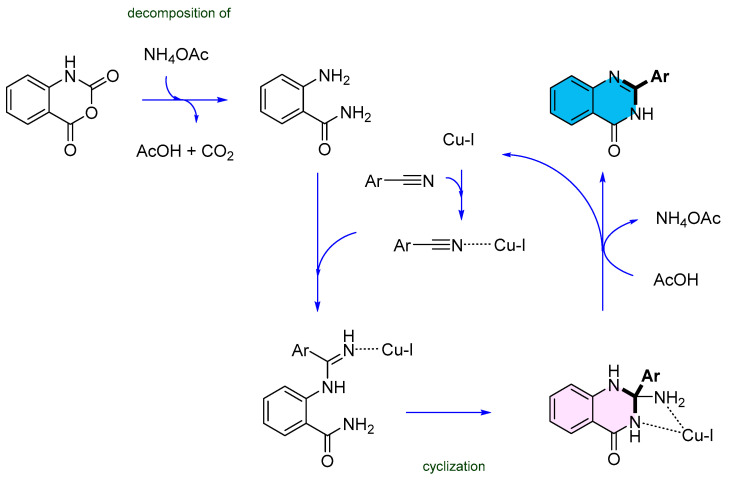
Mechanism of the synthesis of 2-arylquinazolinones proposed by Soheilizad et al. [48].

Anthranilamide, formed in situ from the reaction of isatoic anhydride with ammonia (generated through thermal decomposition of NH_4_OAc) (Scheme 26), condensed with a copper-activated nitrile intermediate, to form the 2-benzimidamidobenzamide intermediate. Subsequent intramolecular cyclization, followed by the liberation of ammonia, ultimately led to the formation of the corresponding 2-arylquinazoline-4(3*H*)-one.

This strategy afforded the target compounds in good-to-excellent yields (70–87%) and additionally tolerated a wide range of electron-donating (e.g., Me, MeO, or 4-NMe_2_) and electron-withdrawing (e.g., Cl, F, NO_2_) substituents, regardless of their position on the aromatic ring of the benzonitriles. Moreover, the reaction of heterocyclic nitriles, in particular, furan-2-carbonitrile or thiophene-2-carbonitrile, with isatoic anhydride and NH_4_OAc provided the desired products in yields of 77% and 81%, respectively.

### 2.3. Ruthenium-Catalyzed Multicomponent Synthesis of Quinazolinones

**Scheme 27:**
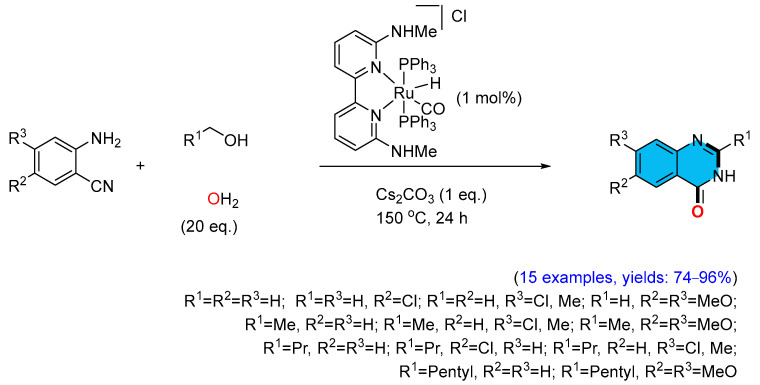
Synthesis of 2-substituted quinazolinones from 2-aminobenzonitriles using aliphatic alcohol–water system [49].

An interesting report on the synthesis of 2-substituted quinazolin-4(3*H*)-ones from 2-aminobenzonitriles using an aliphatic alcohol–water system was presented by Kundu et al. [49]. However, Wu et al. [31] already noted the potential of 2-aminobenzonitriles as convenient and readily available substitutes for 2-aminobenzamides in the metal-catalyzed synthesis of quinazolinones. The main challenge for the authors was to develop an appropriate catalyst for this conversion. Water, used as a green solvent, played a crucial role in the overall process, enabling the hydration of the nitrile to the anthranilamide. Accordingly, the catalyst ought to be stable and effective during the alcohol dehydrogenation step in an aqueous medium. Among several different ruthenium complexes screened—containing a ruthenium hydrochloride carbon monoxide unit and bidentate, functionalized 2,2′-bipyridine ligands—the complex with the *N*6,*N*6′-dimethyl-2,2′-bipyridine-6,6′-diamine ligand exhibited the highest activity (Scheme 27). In addition, various bases were tested (e.g., Cs_2_CO_3_, KO*^t^*Bu, K_2_CO_3_), and it was found that using 1 equivalent of Cs_2_CO_3_ in combination with 20 equivalents of water gave the best results for this transformation.

Based on the presented strategy, a series of various substituted quinazolinones was synthesized from 2-aminobenzonitriles bearing both electron-donating and electron-withdrawing groups using a range of straight-chain alcohols, such as methanol, ethanol, butanol, and hexanol. The desired lactams were obtained in good-to-excellent yields (74–96%).

**Scheme 28:**
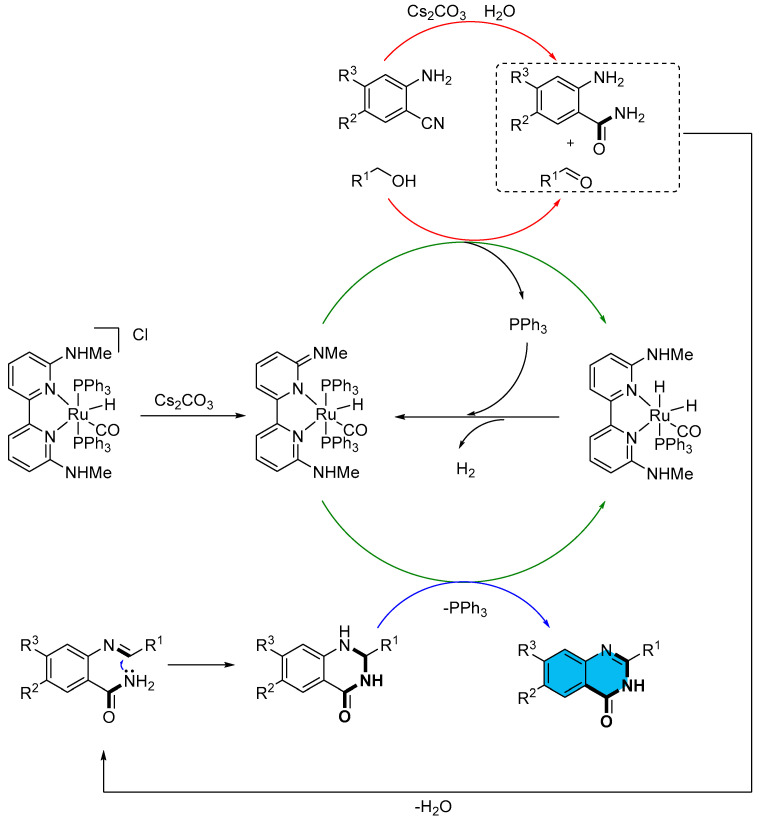
Proposed reaction mechanism for the Ru-catalyzed synthesis of 2-substituted quinazolinones from 2-aminobenzonitriles using aliphatic alcohol–water system [49].

An important issue for the authors was understanding the mechanism of this conversion (Scheme 28). Several experiments were conducted involving the reactions of 2-aminobenzonitrile with methanol-d_4_ in the presence of D_2_O as well as reactions with methanol in the presence of H_2_O^18^. These experiments confirmed that methanol was the source of C-2 carbon, while water was the source of the oxygen atom for the carbonyl group in the final quinazolin-4(3*H*)-one. Moreover, it was demonstrated that the ruthenium complex played a key role in the dehydrogenation of 2,3-dihydroquinazolin-4(1*H*)-one, one of the intermediates on the pathway to quinazolinone. The proposed mechanism for this tandem reaction initially involved the formation of the active catalyst species from a ruthenium precursor complex under basic conditions, which dehydrogenates the alcohol to produce the corresponding aldehyde. Simultaneously, the hydration of 2-aminobenzonitrile furnished 2-aminobenzamide, which then condensed with an aldehyde to form an imine intermediate. Further intramolecular cyclization, followed by dehydrogenation of the resulting 2,3-dihydroquinazolinone, led to the formation of the desired product. Additionally, after each dehydrogenation cycle, a hydrogen molecule was eliminated from the ruthenium dihydride species, thereby regenerating the active catalyst.

### 2.4. Cobalt-Catalyzed Multicomponent Synthesis of Quinazolinones

**Scheme 29:**
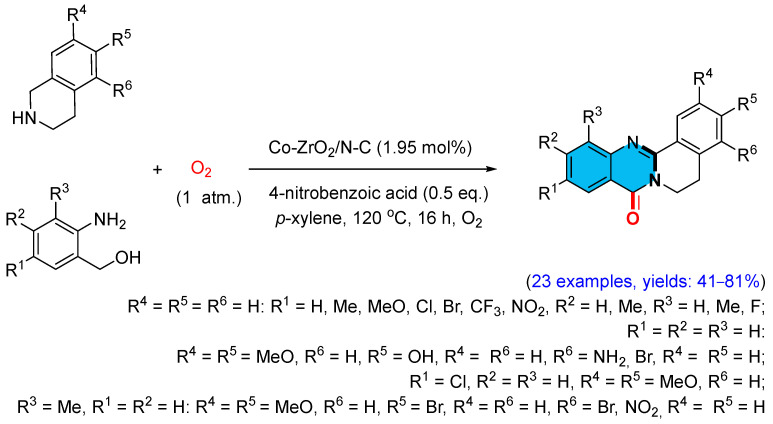
Synthesis of 5,6-dihydro-8*H*-isoquinolino[1,2-b]quinazolin-8-ones [50].

Zhang et al. [50] reported the development of a new cobalt nanocatalyst supported on nitrogen-doped carbon—denoted in Scheme 29 as Co-ZrO_2/_N-C—prepared via an MOF (Metal–Organic Framework) templated method, for the efficient synthesis of ring-fused quinazolinones from readily available cyclic amines (mainly 1,2,3,4-tetrahydroisoquinoline derivatives) and 2-aminoarylmethanols (Scheme 29).

Optimal yields of the desired products were achieved using 1.95 mol% of a cobalt nanocatalyst and molecular oxygen as a green oxidant in the presence of 0.5 eq 4-nitrobenzoic acid as the additive in *para*-xylene at 120 °C. The described methodology featured a broad substrate scope, good functional group tolerance, and high step and atom efficiency. It can be noted that (2-aminophenyl)methanols bearing the electron-donating groups (Me, MeO) afforded relatively higher yields of products compared to those with strong electron-withdrawing ones (Cl, F, Br, CF_3_, NO_2_). Similarly, tetrahydroisoquinolines with electron-donating groups (MeO, OH, NH_2_) provided higher product yields than derivatives bearing electron-deficient groups (Cl, Br, NO_2_). In both cyclic amines and 2-aminoarylmethanols, electron-rich substituents increase the nucleophilicity of the amine nitrogen atom, thereby favoring the coupling steps. The authors also examined the reusability of the developed nanocatalyst. A decrease in conversion in the model reaction was observed in each of the five recycling cycles performed (from, 70%, 60%, 50%, 50% up to 45%). To improve reaction conversion over the reused catalysts, the Co-ZrO_2/_N-C was reactivated at 800 °C for 2 h under an Ar atmosphere for the next reaction cycle. It was found that reactivating the reused catalyst contributed to an increase in yields; however, as the number of cycles increased, a decline was still observed in the reaction conversion.

**Scheme 30:**
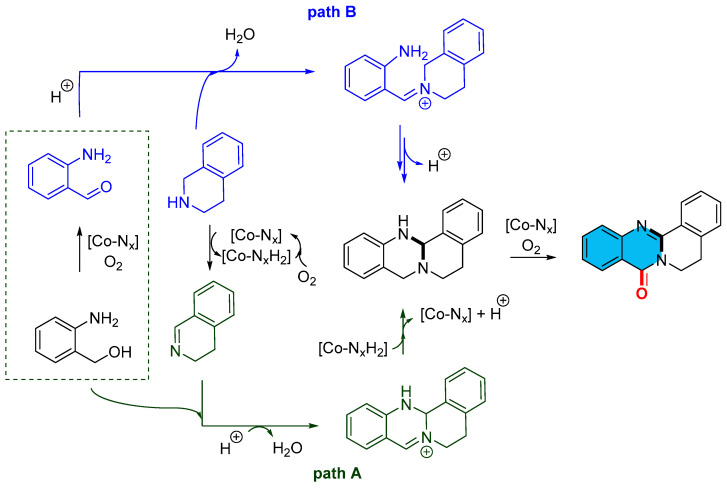
Suggested mechanism of the Co-catalyzed synthesis of ring-fused quinazolinone derivatives [50].

Mechanistic studies, based on several experiments, suggested two possible pathways for this transformation, as depicted in Scheme 30. Regardless of the chosen reaction pathway, the key intermediate was the 5,8,13,13a-tetrahydro-6*H*-isoquinolino[1,2-b]quinazoline derivative, which, after oxidation, yielded the target quinazolinone.

### 2.5. Iridium-Catalyzed Multicomponent Synthesis of Quinazolinones

**Scheme 31:**
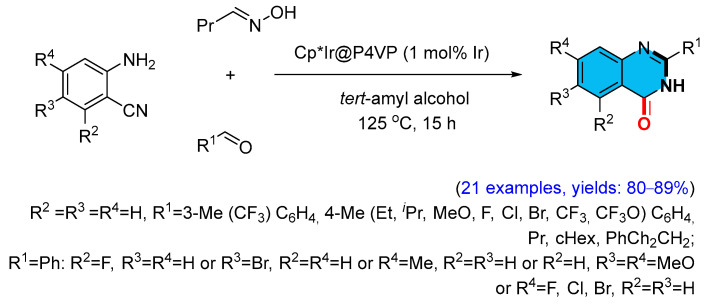
Cp*Ir@P4VP-catalyzed synthesis of 2-substituted quinazolinone from *o*-aminobenzonitrile [51].

In 2021, Li et al. [51] reported the synthesis and application of a linear organic polymer-supported iridium complex (Cp*Ir@P4VP), prepared by the coordinative immobilization of [Cp*IrCl_2_]_2_ (pentamethylcyclopentadienyliridium(III) chloride, dimer) on poly(4-vinylpyridine) (P4VP), which served as a recyclable heterogeneous catalyst for the efficient synthesis of quinazolin-4(3*H*)-ones (Scheme 31). Several years earlier, the same authors [52] reported a similar strategy for the direct preparation of quinazolinones via an iridium-catalyzed one-pot sequential (stepwise addition of reactants) selective hydration combined with acceptorless dehydrogenation from *o*-aminobenzonitriles. Although this procedure was attractive due to its high atom economy, use of readily available raw materials, and minimal energy and chemical consumption, its practical application was limited by the non-recyclability of the homogeneous catalyst.

Initially, the coupling of *o*-aminobenzonitrile, *n*-butylaldoxime (1.2 eq. used as a water surrogate), and benzylaldehyde (1.2 eq.) was carried out in the presence of 1 mol % Cp*Ir@P4VP, in *tert*-amyl alcohol at 110 °C for 15 h, affording the desired 2-phenylquinazolin-4(3*H*)-one in an 80% yield. Increasing the reaction temperature to 125 °C resulted in an improved yield of 93%. In addition, alternative solvents including 1,4-dioxane, THF, 2-MeTHF, and toluene were evaluated, but product yields ranged from 43 to 87%.

The catalyst exhibited a broad substrate scope, tolerating various substituted benzaldehyde-bearing groups such as 3-Me (CF_3_), 4-Me (Et, *^i^*Pr), and 4-MeO (OCF_3_, CF_3_, Br or F), as well as aliphatic aldehydes including phenylpropylaldehyde, butyraldehyde, and cyclohexanecarbaldehyde, affording the corresponding products in 78–89% yields. The system was also compatible with substituted *o*-aminobenzonitriles, bearing Me, MeO, or halogen groups (F, Br, Cl), producing the target quinazolinones with yields of 80–87%.

**Scheme 32:**
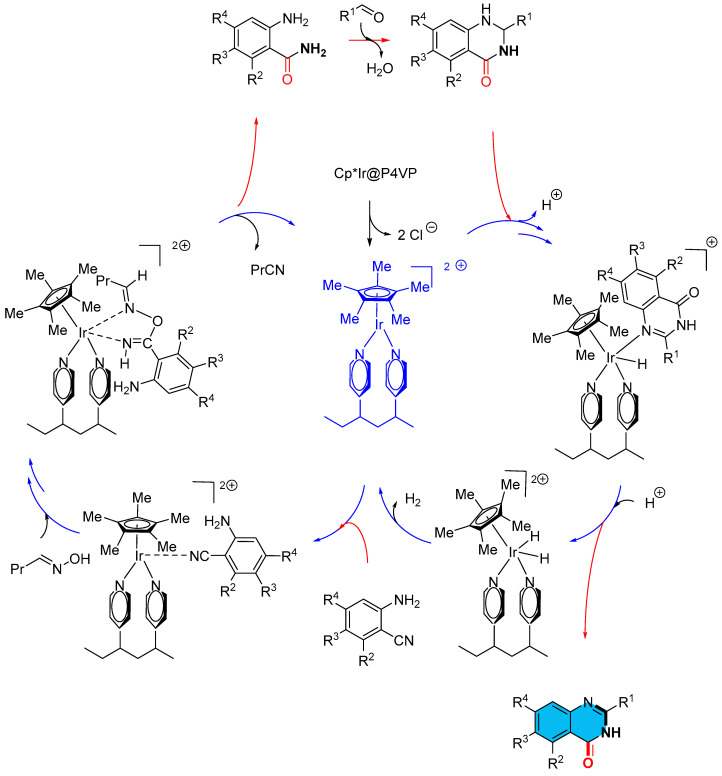
Proposed mechanism reaction for the Ir-catalyzed synthesis 2-substituted quinazolinones [51].

The catalyst’s effectiveness was also confirmed in a gram-scale, two-step synthesis of the natural product *Schizocommunin* via the formation of 2-methylquinazolinone under standard reaction conditions followed by the reaction with isatin in the presence of AcOH. Moreover, the catalyst was recycled five times without an obvious decrease in catalytic activity. Based on the results of several mechanistic experiments and the literature data, a dual catalytic cycle was proposed (Scheme 32). In the first cycle, *o*-aminobenzonitrile underwent hydration to form *o*-aminobenzamide, which proceeded in the presence of *n*-butylaldoxime and was facilitated by the iridium-catalytically active species. The condensation of antranilamide with an aldehyde led to the formation of 2,3-dihydroquinazolinone. The second cycle involved the deprotonation and coordination of 2,3-dihydroderivative to the unsaturated iridium species. Subsequently, the *β*-hydrogen elimination and protonation yielded the desired quinazolin-4(3*H*)-one along with iridium dihydride species. The release of hydrogen gas from iridium dihydride regenerated the active catalytic species.

## 3. Strategies for the Synthesis of Quinazolinones Involving Isatoic Anhydride (Scheme 33)

**Scheme 33:**
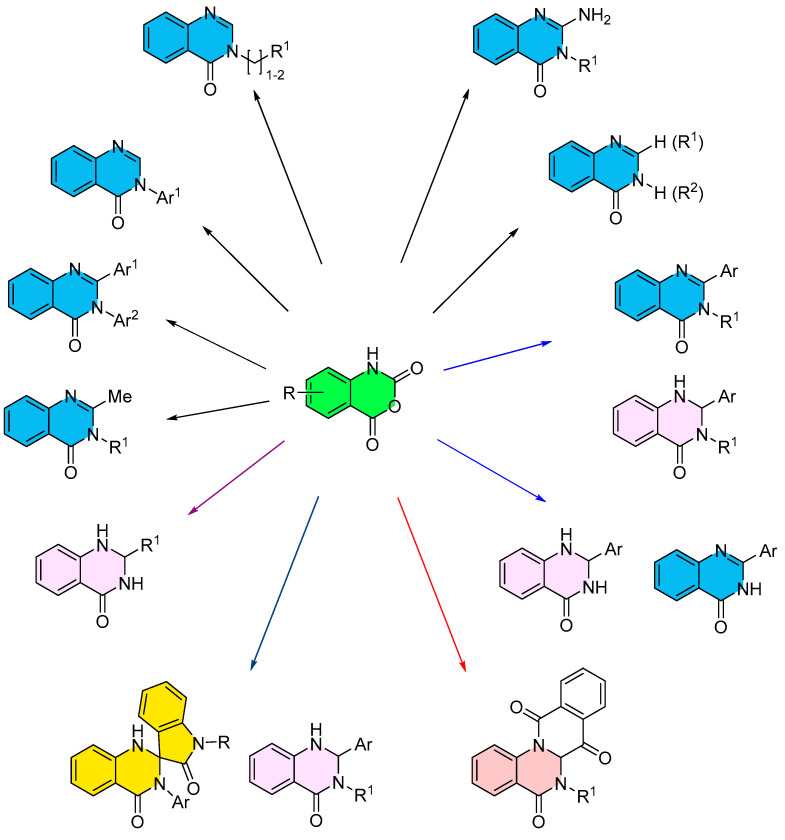
Strategies for the synthesis of quinazolinones involving isatoic anhydride.

One of the more widely used strategies for obtaining the quinazolinone skeleton involves the use of isatoic anhydride as a key building block (Scheme 33). Isatoic anhydride is employed in various multicomponent reactions with amines and aldehydes, ortho esters, or diketones, providing access to a broad range of substituted quinazolin-4(3*H*)-ones and 2,3-dihydroquinazolin-4(1*H*)-ones, often under mild and environmentally friendly conditions. Selected examples illustrating this approach are presented below.

**Scheme 34:**
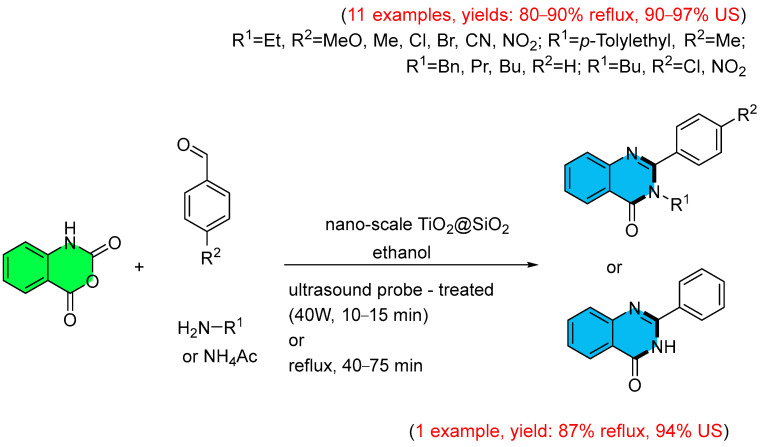
Ultrasound-irradiation-assisted synthesis of 2,3-disubstituted quinazolinones [53].

The introduction of ultrasound irradiation (US) as a highly efficient and environmentally friendly activation method for the one-pot construction of 2,3-disubstituted quinazoline-4(3*H*)-ones was realized by Fadaeian et al. [53]. They reported the fabrication and application of a nano-scale TiO_2_@SiO_2_ composite as a robust and reusable heterogeneous catalyst for the efficient synthesis of 2,3-disubstituted quinazoline-4(3*H*)-ones (Scheme 34). The catalyst was employed in a three-component reaction involving isatoic anhydride, various aromatic aldehydes, and amine components (aryl and aliphatic amines, or ammonium acetate) in ethanol as a green solvent.

The authors compared two synthetic methods: conventional reflux and ultrasound-probe-assisted synthesis (40 W). The ultrasound method, benefiting from cavitation effects, proved more efficient, delivering excellent yields (90–97%) within a short reaction time (up to 10 minutes, compared to several hours under reflux) and requiring only a low catalyst amount (12 mg). The TiO_2_@SiO_2_ nanocatalyst demonstrated excellent reusability over seven cycles with negligible loss in catalytic activity. The nano-scale catalyst played a vital role as a Lewis acid. The acidic sites of the nano-scale TiO_2_@SiO_2_ composite bound to the electron pair of the oxygen atom of the carbonyl group and the π electrons of the imine C=N group, activating both compounds and facilitating, on the one hand, the nucleophilic attack of the amine on the isatoic anhydride, and on the other hand, the intramolecular cyclization of the imine to form 2,3-dihydroquinazolinone framework. The final oxidation step yielded the 2,3-disubstituted quinazoline-4(3*H*)-one.

**Scheme 35:**
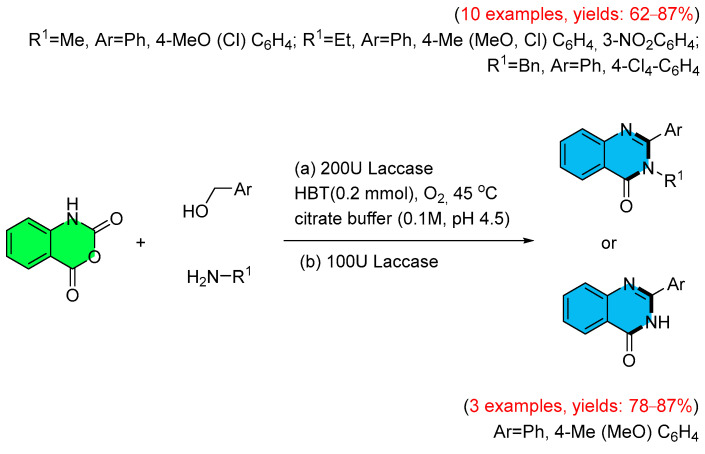
Synthesis 2-arylquinazolinones in the reactions of isatoic anhydride with substituted benzyl alcohols, and alkyl amines [54].

Building on previous research and leveraging the well-known ability of laccase derived from *Trametes versicolor* to catalyze the oxidation of benzyl alcohols to benzaldehydes, Faramarzi [54] (Scheme 35) presented a novel, environmentally friendly, and efficient strategy for synthesizing 2-arylquinazolin-4(3*H*)-ones, involving a three-component, one-pot reaction between isatoic anhydride and various substituted benzyl alcohols and alkyl amines in a citrate buffer (pH 4.5) as a green solvent at 45 °C.

The reaction proceeded through the initial reaction of isatoic anhydride with an amine to produce an aminobenzamide intermediate, which subsequently underwent cyclocondensation with an aldehyde—generated in a laccase-catalyzed oxidation step from the corresponding benzyl alcohol—to yield 2,3-dihydroquinazolin-4(1*H*)-one. Further laccase-mediated oxidation (in the presence of *N*-hydroxybenzotriazole (HBT) under O_2_) of this intermediate produced the final quinazolinone product. This approach avoided the use of volatile aldehydes, produced only water as a by-product, and afforded quinazolinones in good-to-excellent yields (62–87%). The yields of the produced products were affected by the nature of the substituent on the benzene ring of benzyl alcohol. In general, benzyl alcohols with an electron-withdrawing group exhibited lower oxidative reactivity to the corresponding aldehydes and finally afforded the target lactams in poor yields.

**Scheme 36:**
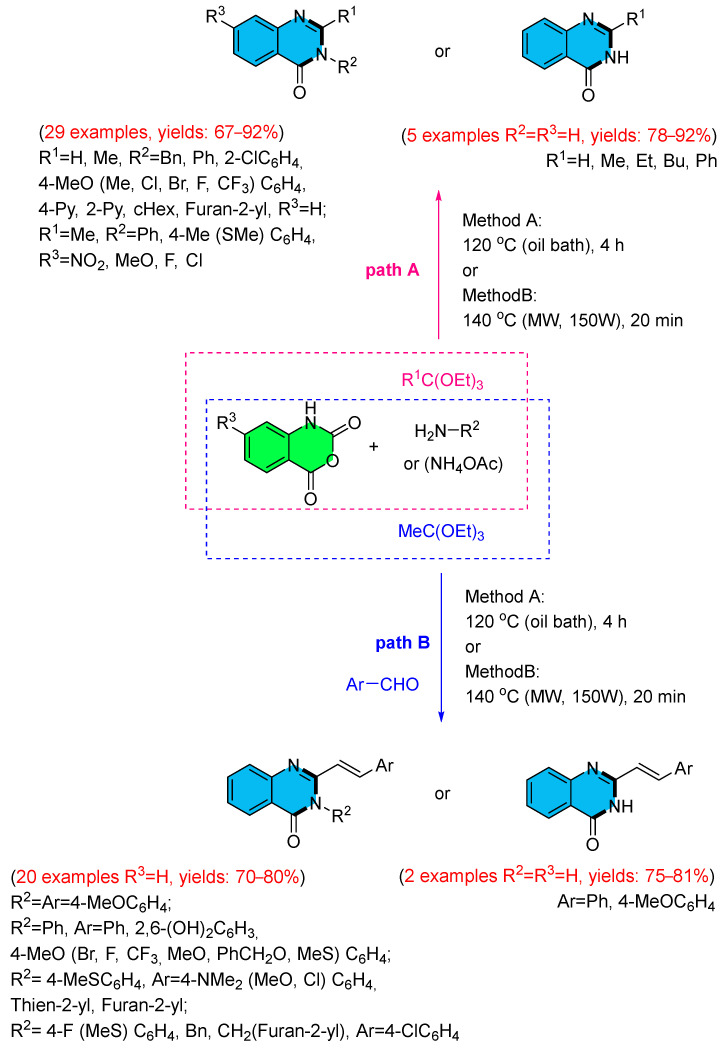
Synthesis of multi-substituted quinazolinones [55].

Synthesis of multi-substituted quinazolinone derivatives, including 2- or 3-monosubstituted, 2,3-disubstituted, and 2,3,6-trisubstituted compounds, through three-component, one-pot reactions was reported by Chakraborti [55] (Scheme 36, path A).

**Scheme 37:**
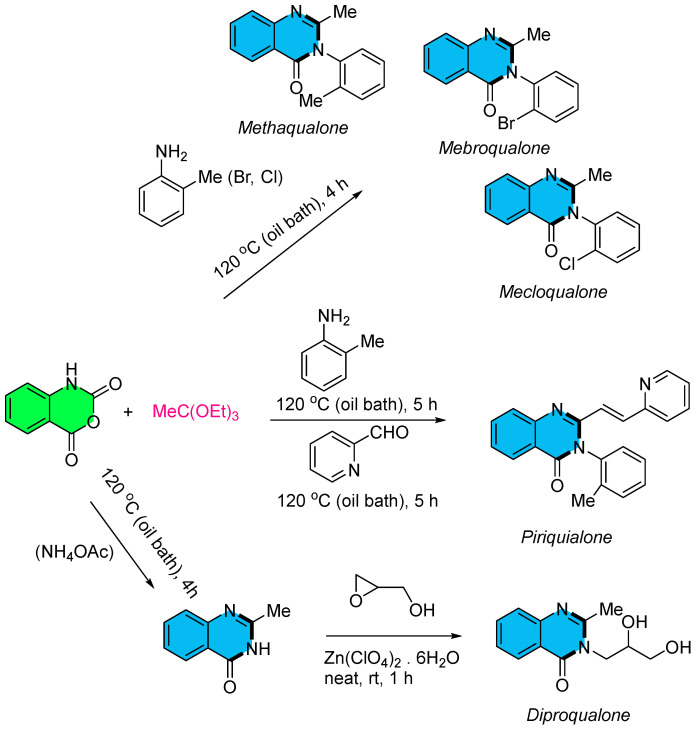
Synthesis of Methaqualone, Mebroqualone, Mecloqualone, Piriquialone, and Diproqualone [55].

The solvent- and catalyst-free protocol involved the condensation of various isatoic anhydrides, amines or ammonium acetate (NH_4_OAc), and ortho esters, such as triethyl orthoacetate or triethyl orthoformate under conventional (120 °C, oil bath, 4 h) or microwave heating (140 °C, 20 min), affording the corresponding quinazolin-4(3*H*)-ones in excellent yields (up to 92%). Moreover, a wide range of substituted isatoic anhydrides and aryl, heteroaryl, arylalkyl, alkyl, and allyl amines were compatible with this method, leading to the target compounds. An exception was observed for amines bearing electron-withdrawing substituents in the benzene ring, which resulted in lower yields of the corresponding 2,3-disubstituted lactams. Furthermore, 2-methylquinazolinones served as starting materials for the preparation of 2-styrylquinazolinones by the reaction with various aldehydes through a two-step, one-pot procedure without the need for acid or base in the condensation step (Scheme 36, path B). The synthetic utility of the described methodologies was demonstrated in the synthesis of several CNS depressants, such as Methaqualone, Mebroqualone, Mecloqualone, Piriquialone, and Diproqualone, in good yields (Scheme 37).

**Scheme 38:**
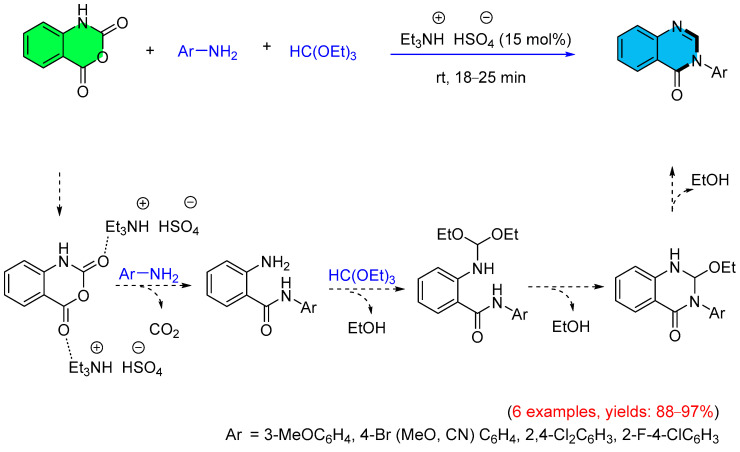
Synthesis of 2-aryl quinazolinones [56].

In 2021, Awasthi [56] demonstrated the potential of a simple ammonium-based ionic liquid [Et_3_NH]^+^[HSO_4_]^–^ as an efficient catalyst for the synthesis of a series of 3-aryl quinazolinones. The protocol employed isatoic anhydride, variously substituted anilines, and triethyl orthoformate under green, mild, and high-yielding conditions (Scheme 38). Reactions were carried out at room temperature, under solvent-free conditions, and completed within 18–25 min. Anilines bearing electron-donating substituents afforded products in excellent yields, whereas those with electron-withdrawing groups led to moderate yields. It was observed that increasing the amount of ionic liquid to 15 mol% significantly improved product yields. However, further increasing the amount to 30 mol% did not result in any additional improvement. Similarly, raising the temperature or extending the reaction time had no notable effect on the reaction outcome. The ionic liquid acted as an activator of the carbonyl group of isatoic anhydride, facilitating the nucleophilic attack of aniline and the subsequent release of carbon dioxide, leading to the formation of the anthranilamide intermediate. In the next stage, the amine group underwent a nucleophilic attack on triethyl orthoformate, followed by the elimination of ethanol molecules and, finally, intramolecular cyclization to form the quinazolinone ring.

**Scheme 39:**
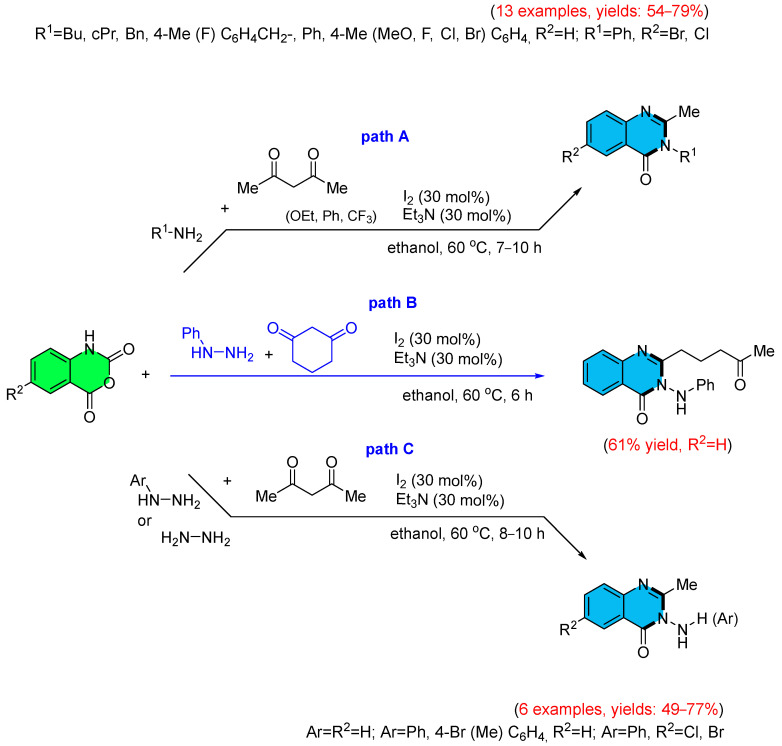
I_2_-catalyzed synthesis of 2,3-disubstituted quinazolinones [57].

Pramanik et al. [57] demonstrated the construction of 2,3-disubstitutedquinazolinone scaffolds with moderate-to-good isolated yields (49–79%) from a one-pot, two-step, three-component reaction between various isatoic anhydrides, amines (Scheme 39, path A) or hydrazine (arylhydrazines) (Scheme 39, path B, C), and both cyclic and acyclic 1,3-diketones, as well as *β*-keto esters, in the presence of a catalytic system containing molecular I_2_ and Et_3_N in an optimal ratio of 30 mol% each under mild, open-air conditions (ethanol, 60 °C).

Attempts to use other catalysts, such as FeCl_3_, CuBr, AlCl_3_, I_2_/NaHCO_3_, or Et_3_N in various molar ratios in DMF or ethanol, remained ineffective in forming the desired products. Moreover, when 30 mol% I_2_ in ethanol was used without Et_3_N, the quinazolinone derivative was obtained in only a 30% yield. On the other hand, when the reaction was performed only in the presence of 30 mol% Et_3_N without iodine, the antranilamide intermediate was formed in a very high yield as the sole product, without any trace of the quinazolinone derivative. These results suggest that the target quinazolinone was derived from the antranilamide derivative and was formed via an intermolecular condensation with acetylacetone, followed by oxidative C-C bond cleavage. The method was effective in the presence of aromatic amines containing both electron-donating and electron-withdrawing substituents. Also, various aliphatic amines, such as butylamine or cyclopropylamine, produced the desired quinazolinone derivatives in satisfactory yields. Interestingly, this strategy was found to be effective with hydrazine and aromatic hydrazines, successfully leading to the analogous 2-methyl-3-(arylamino)quinazolinones. In addition, a preliminary biological study showed that 3-(4-bromophenylamino)-2-methylquinazolin-4(3*H*)-one is cell-permeable and can be used as a fluorescent probe for bio-imaging of human cervical cancer (HeLa) cells.

**Scheme 40:**
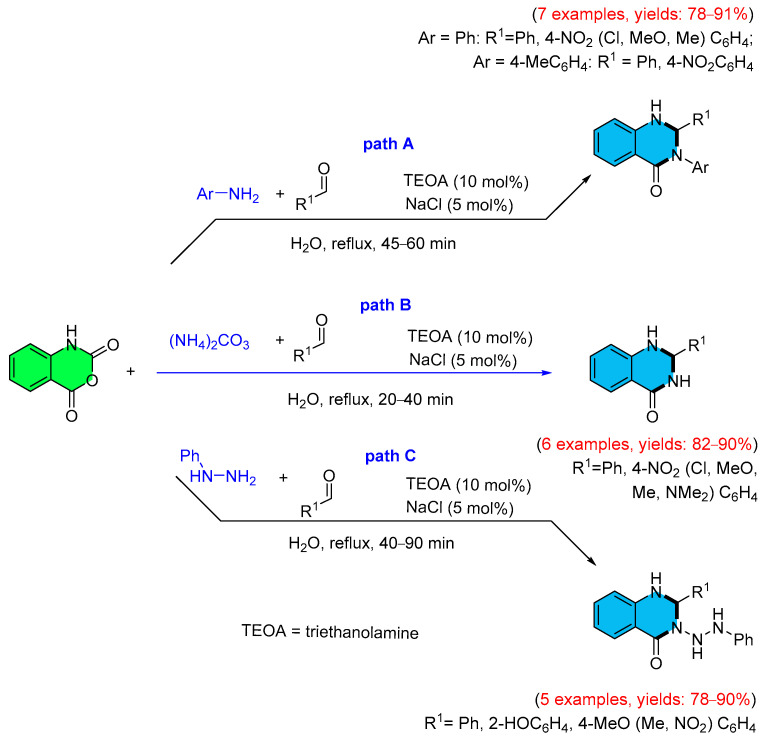
Synthesis of 2,3-dihydroquinazolinones [58].

In turn, Sarda et al. [58] described an eco-friendly method for the synthesis of 2,3-dihydroquinazolinone derivatives via a one-pot, three-component reaction catalyzed by triethanolamine (TEOA, 10 mol%) in the presence of sodium chloride (5 mol%) in aqueous media under reflux conditions (Scheme 40). Interestingly, the reaction did not proceed at room temperature, and increasing the temperature to 60 °C resulted in only a slight improvement in product yield, indicating the limited thermal sensitivity of the process. Water was selected as the reaction medium due to its environmentally benign nature, non-toxicity, and potential to reduce energy consumption and pollution. The key starting materials in this protocol included an aromatic aldehyde, isatoic anhydride, and an amine component (such as phenylhydrazine, ammonium carbonate, or an aromatic amine). TEOA acted as a nonionic surfactant, and NaCl enhanced the surfactant’s micellar properties and increased hydrophobic interactions, leading to higher yields and shorter reaction times. The TEOA/NaCl system demonstrated excellent selectivity, not only promoting the formation of the target compound but also enabling a cleaner transformation pathway. For comparison, the catalytic activity of ethanolamine and diethanolamine was also investigated. Diethanolamine provided better yields than ethanolamine; however, it was not as effective as TEOA.

**Scheme 41:**
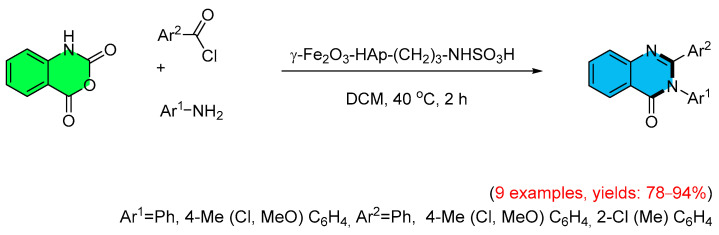
Synthesis of 2,3-diarylquinazolinones by the reaction of isatoic anhydride with acylchlorides and anilines in the presence of [γ-Fe_2_O_3_-HAp-(CH_2_)_3_-NHSO_3_H] [59].

Dadgar et al. [59] (Scheme 41) reported the efficient synthesis of 2,3-diarylquinazolin-4(3*H*)-ones by the reaction of isatoic anhydride with benzoyl chlorides and anilines in the presence of a highly effective and magnetic-separable heterogeneous catalyst, propylsulfamic-acid-functionalized magnetic hydroxyapatite nanoparticle [γ-Fe_2_O_3_-HAp-(CH_2_)_3_-NHSO_3_H] (0.75 mol%), under mild conditions (DCM 40 °C) and short reaction times (2 h). Amines and acyl halides with an electron-donating (Me, MeO) or electron-withdrawing (Cl) groups tolerated the reaction conditions well, giving the corresponding quinazolinone in acceptable yields. Surprisingly, the reaction did not proceed in the absence of [γ-Fe_2_O_3_-HAp-(CH_2_)_3_-NHSO_3_H]. On the other hand, the use of larger amounts of catalyst did not result in a significant improvement in reaction efficiency, nor did increasing the reaction temperature or using DMF as a solvent. Moreover, the catalyst could be easily recovered using an external magnet and reused up to three times without significant loss of activity.

**Scheme 42:**
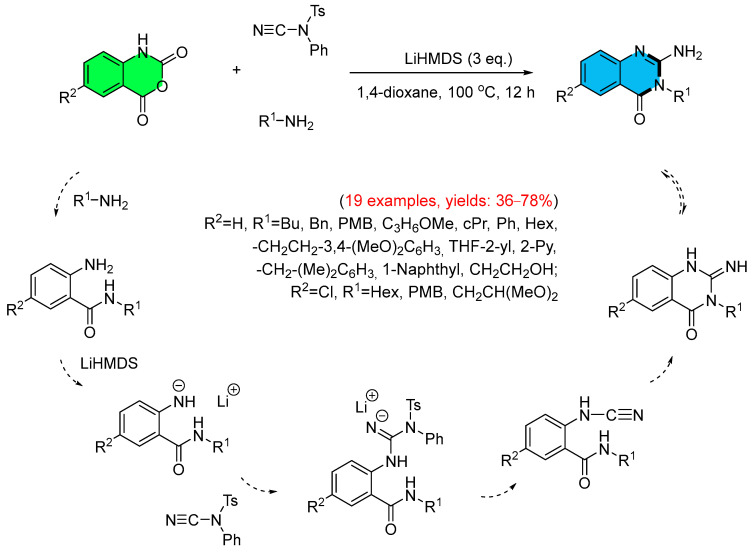
Synthesis of 2-amino-3-substitutedquinazolines [60].

Raghunadh et al. [60] explored the synthesis of 2-amino-3-substitutedquinazolines (Scheme 42) from the reaction of isatoic anhydride with aromatic, heteroaromatic, or aliphatic amine and a stable, non-toxic, electrophilic cyanating agent, *N*-cyano-4-methyl-*N*-phenylbenzenesulfonamide, in a one-pot process. As it turned out, both the base and solvent used had a significant impact on the reaction outcomes and final yields. Among the tested bases (K_2_CO_3_, DBU (1,8-diazabicyclo[5.4.0]undec-7-ene), DABCO (1,4-diazabicyclo[2.2.2]octane), Et_3_N, Cs_2_CO_3_, and LiHMDS (lithium hexamethyldisilazane)), the best result was obtained when the reaction was carried out in the presence of LiHMDS in 1,4-dioxane as a solvent at 100 °C, which proved to be the most effective solvent for this reaction. Other solvents, including DMSO, DMF, THF, acetonitrile, or toluene, were less effective. A detailed reaction mechanism (lower part of Scheme 42) first involved the nucleophilic attack of a primary amine on the carbonyl group of isatoic anhydride followed by ring opening and subsequent decarboxylation, leading to antranilamide. In the next step, the deprotonation of aromatic amine by a base enabled the nucleophilic attack on the nitrile group, yielding an imine intermediate. The elimination of the *N*-phenyl tosyl group, followed by cyclization and tautomerization, finally led to the formation of the desired quinazolinone.

**Scheme 43:**
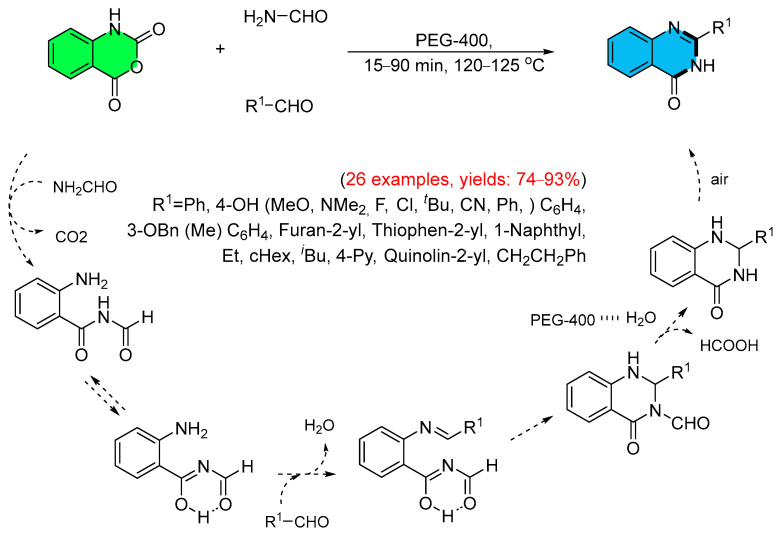
Synthesis of 2-substituted quinazolinones [61].

The diverse pharmacological activities of quinazolin-4(3*H*)-one-based alkaloids, such as Luotonin B and E and Bouchardatine, inspired Pal et al. [61] to develop a facile and catalyst-free method for the construction of a quinazolin-4(3*H*)-one skeleton (Scheme 43). The methodology afforded various 2-substituted quinazolinone derivatives in good-to-excellent yields (74–93%) via a three-component reaction of isatoic anhydride, aldehydes, and formamide as an efficient ammonia precursor. The reaction was carried out in PEG-400, a high-boiling, inexpensive, non-toxic, and effective solvent, under open-air conditions. PEG-400 proved to be more effective than other commonly used organic solvents, such as MeOH, EtOH, BuOH, CHCl_3_, toluene, or 1,4-dioxane, providing a higher reaction temperature and shorter reaction time.

The reaction proceeded well with the aliphatic (alkyl, cycloalkyl, alkylaryl) aldehydes and aromatic aldehydes bearing various substituents on the aromatic ring (e.g., hydroxy, alkoxy, amine, halogens, phenyl, cyano or benzyloxy) as well as heteroaryl aldehydes (including derivatives of furan, pyrrole, pyridine, thiophene, quinoline, or indole). Interestingly, the reaction also proceeded efficiently in the absence of an aldehyde when it was carried out for 90 min, affording the unsubstituted quinazolin-4(3*H*)-one in an 81% yield. This clearly demonstrates the usefulness of formamide as an effective ammonia surrogate in the synthesis of quinazolin-4(3*H*)-ones.

**Scheme 44:**
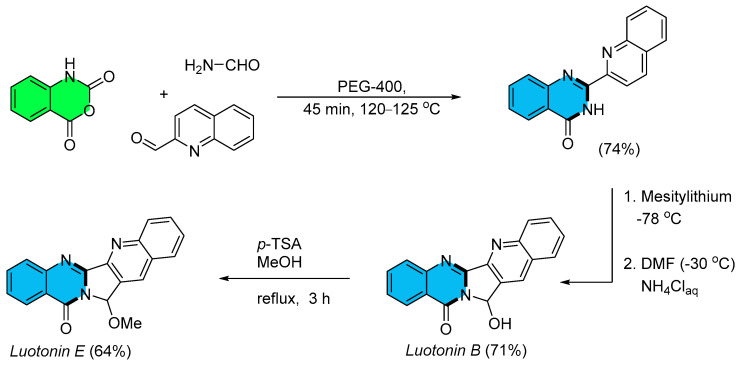
Synthesis of alkaloids Luotonin B and E [61].

The probable reaction mechanism (lower part of Scheme 43) initially involved the formation of a 2-amino-*N*-formylbenzamide intermediate, which easily tautomerized to its enol via the formation of an intramolecular hydrogen bond, producing a six-membered cyclic form. In the next step, the amino group reacted with the aldehyde, followed by intramolecular cyclization of an imine, to give 4-oxo-1,4-dihydroquinazoline-3(2*H*)-carbaldehyde. The cleavage of the N–C bond in the -N-CH=O moiety, aided by H_2_O at an elevated temperature, yielded 2,3-dihydroquinazolin-4(1*H*)-one, which, upon further oxidation in the presence of air, afforded the final quinazolinone. The significant acceleration of the reaction in PEG-400 was likely caused by its participation in the deformylation stage by increasing the nucleophilicity of the water molecule through the formation of a hydrogen bond between the PEG-400 oxygen atom and the hydrogen atom of H_2_O, as well as in the aerobic oxidation at an elevated reaction temperature. This methodology was successfully extended to the synthesis of several alkaloids, e.g., Luotonin B and E (Scheme 44).

**Scheme 45:**
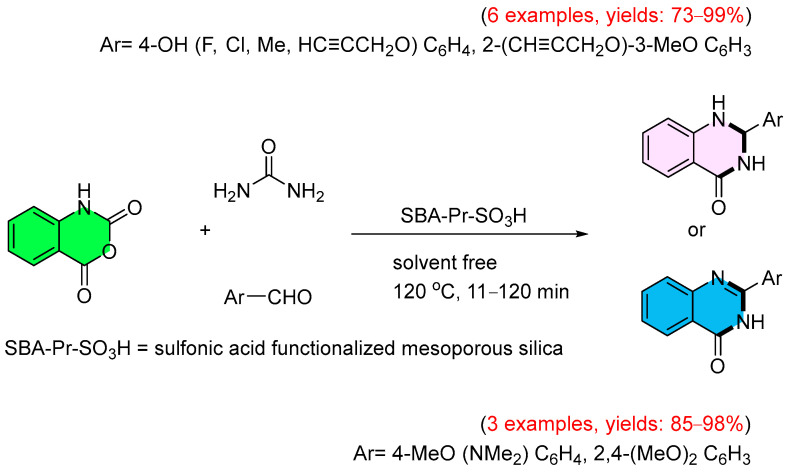
Synthesis of 2,3-disubstituted quinazolinones and 2,3-dihydroquinazolinones [62].

Ziarani et al. [62] (Scheme 45) presented the catalytic activity of sulfonic-acid-functionalized mesoporous silica (SBA-Pr-SO_3_H) in the one-pot three-component synthesis of quinazolinones, starting from isatoic anhydride, various substituted aromatic aldehydes, and urea under solvent-free conditions at 120 °C. SBA-Pr-SO_3_H was obtained by reacting SBA-15 with 3-mercaptopropyltriethoxysilane, followed by oxidation of the grafted thiol groups by the use of hydrogen peroxide. 4-OH, 4-F, 4-Cl, 4-Me, and propargyl ether substituents yielded non-oxidized 2,3-dihydroquinazolin-4(1*H*)-one, while 4-NMe_2_, 4-OMe, and 2,4-(MeO)_2_ benzaldehyde derivatives produced oxidized 3-arylquinazolinones under an air atmosphere.

**Scheme 46:**
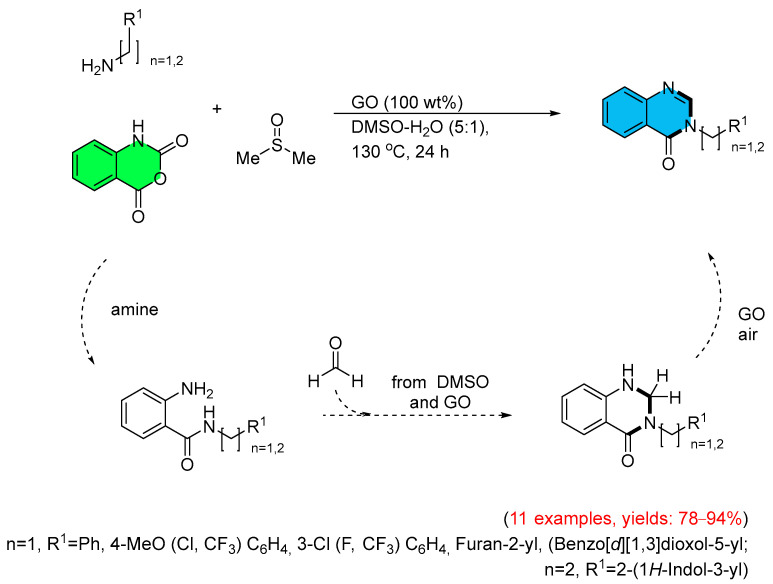
Graphene-oxide-promoted MCR for the synthesis of 3-substituted quinazolinones [63].

Kumari et al. [63] described a graphene oxide (GO, 100 wt%)-promoted reaction for the synthesis of 3-substituted quinazolinone derivatives from readily available isatoic anhydride and amines in a DMSO–water (5:1) solvent mixture at 130 °C for 24 h (Scheme 46). It is worth noting that the reaction did not proceed at temperatures below 130 °C. Moreover, dimethyl sulfoxide served a dual role as both a solvent and a source of formaldehyde generated in situ for the annulation process. GO exhibited superior catalytic activity compared to several other tested carbocatalysts, including reduced GO (rGO), carbon black, and nitrogen-doped reduced graphene oxide (N-rGO). No reaction product was observed when graphite, r-GO, and N-rGO were used. Furthermore, graphene oxide could be reused up to three times with minimal loss of activity (87, 81, and 72% yield, respectively). However, in the fourth run, the activity decreased drastically, yielding only 38% of the quinazolinone product.

A wide range of benzyl amines bearing electron-donating or electron-withdrawing groups on the phenyl ring reacted well with DMSO and isatoic anhydride to produce various quinazolinones. However, benzyl amines with electron-donating groups on the phenyl ring afforded higher yields of the products (74–84%) than those with electron-withdrawing groups (64–77%). Furthermore, amines containing a heterocyclic ring, such as furan-2-ylmethanamine and (1*H*-indol-3-yl)ethan-1-amine, tolerated the reaction conditions well.

Much attention has also been devoted to methods for synthesizing 2,3-dihydroquinazolin-4(1*H*)-ones and spirooxindole derivatives due to their potential biological and pharmaceutical properties, including, analgesic, diuretic, and anticancer activities. Additionally, 2,3-dihydroquinazolinones have found applications as plant growth regulators. Moreover, these compounds can easily be oxidized to their quinazolin-4(3*H*)-one analogues [64]. On the other hand, the spirooxindole ring system is a widely occurring structural motif present in many pharmaceuticals and natural products, including cytostatic alkaloids such as Spirotryprostatins A and B [65].

The following examples illustrate the applicability of multicomponent reactions to the synthesis of 2,3-dihydroquinazolin-4(1*H*)-ones.

**Scheme 47:**
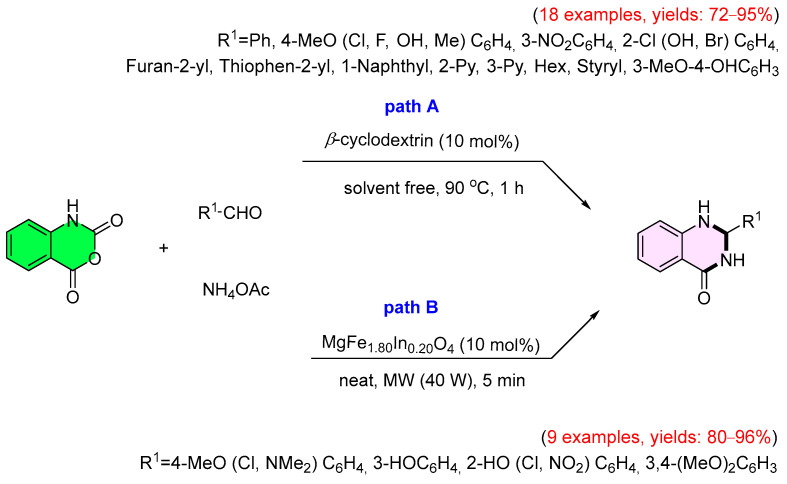
Synthesis 2-substituted 2,3-digydroquinazolinones [66,67].

In 2021, Ghosh et al. [66] developed a highly convenient and eco-friendly approach for the preparation of 2-substituted-2,3-dihydroquinazolin-4(1*H*)-ones in excellent yields (72–95%) (Scheme 47, path A). Their protocol used isatoic anhydride, aromatic (heterocyclic or aliphatic) aldehyde, ammonium acetate as a source of ammonia, and a bio-based supramolecular catalyst, *β*-cyclodextrin, under solvent-free conditions. The novelty of this approach lies in replacing toxic and expensive metal catalysts with an environmentally friendly and inexpensive organocatalyst. This may lead to increased interest in this type of strategy from the pharmaceutical industry. The reactions with various aromatic aldehydes bearing electron-withdrawing and electron-donating groups afforded the desired products in excellent yields; however, slightly better results were obtained with benzaldehydes bearing electron-withdrawing substituents. Similarly, aliphatic aldehyde, as well as five- and six-membered heteroaromatic aldehydes, produced dihydro derivatives with excellent yields. *β*-Cyclodextrin’s ability to form host–guest complexes enhanced the activation of both isatoic anhydride and aldehyde substrate, thereby facilitating the reaction. The reaction proceeded through the formation of a 2-aminobenzamide intermediate, which subsequently condensed with the activated aldehyde, to yield the dihydroquinazolinone framework.

Similarly, Naik et al. [67] (Scheme 47, path B) reported the synthesis of 2-aryl 2,3-dihydroquinazolinones using isatoic anhydride, aromatic aldehyde, and ammonium acetate under solvent-free conditions. The reactions were carried out in the presence of indium-doped magnesium ferrite nanoparticle as a catalyst (MgFe_1.80_In_0.20_O_4_, 10 mol%) under microwave irradiation for 5 min. The use of nanoparticles enhanced the reaction rate and allowed for easy separation and recycling of the catalyst due to its magnetic properties. However, a slight decrease in yield (10%) was observed during recyclability studies over three cycles. The method provides excellent yields (often above 90%) for a broad range of substituted aromatic aldehydes.

Further examples of the synthesis of 2,3-dihydroquinazolinone derivatives focus on the use of water as a green solvent and non-toxic catalysts such as taurine, L-proline, or the surfactant Triton X-100.

**Scheme 48:**
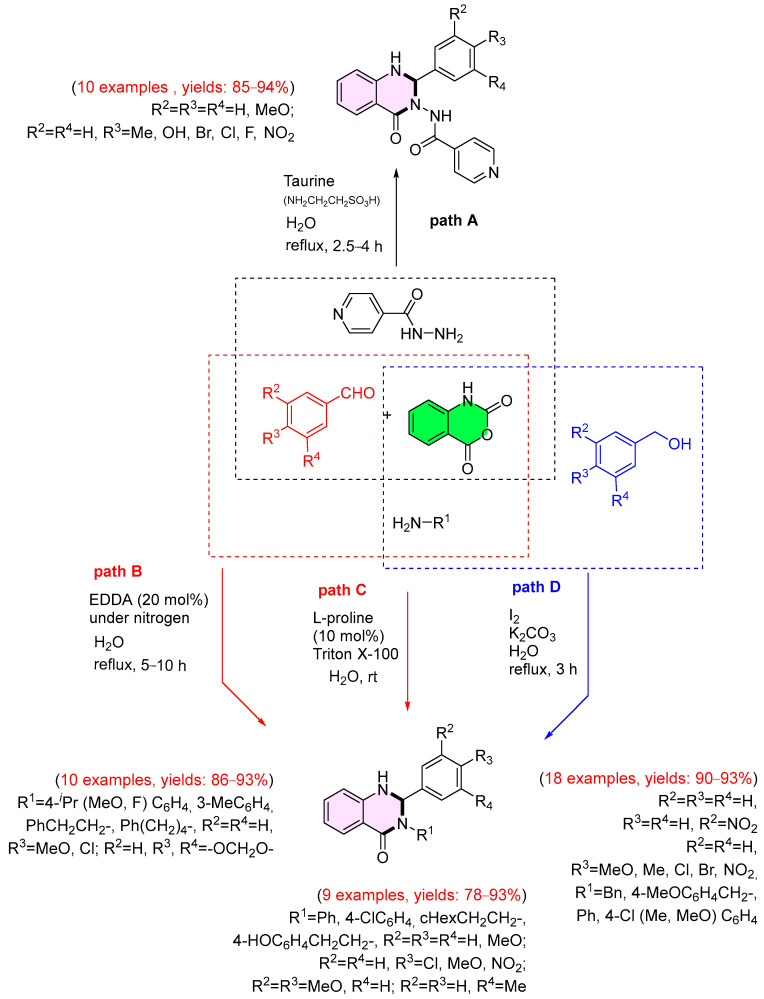
Synthesis of 2,3-disubstituted 2,3-dihydroquinazolinones [68,69,70,71].

In 2020, Chate et al. [68] (Scheme 48, path A) demonstrated a three-component, one-pot synthesis of *N*-(2-aryl-4-oxo-1,4-dihydroquinazolin-3(2*H*)-yl)isonicotinamides from isatoic anhydride, isoniazid, and various aromatic aldehydes using water as a green solvent. The reaction was catalyzed by 2-aminoethanesulfonic acid (taurine), a non-toxic, recyclable, and bifunctional donor–acceptor heterogeneous organocatalyst. The aldehydes used included benzaldehydes substituted with Me, MeO, Cl, Br, F, OH, and NO_2_ groups, as well as thiophene-2-carbaldehyde and 1*H*-indole-3-carbaldehyde. All of the selected aldehydes produced the desired products in excellent yields ranging from 85% to 94%. From a mechanistic point of view, taurine activated the isatoic anhydride, facilitating a nucleophilic attack by the isoniazid. In parallel, taurine also promoted the formation of an imine intermediate via the reaction between an aldehyde and *N*’-(2-aminobenzoyl)isonicotinohydrazide.

In turn, Tripathi [69] (Scheme 48, path C) reported a synthesis of 2,3-disubstituted 2,3-dihydroquinazolinones in aqueous medium, mediated by the surfactant Triton X-100 and the organocatalyst L-proline. The reaction employed variously substituted aromatic aldehydes (e.g., NO_2_, Cl, MeO, Me) as well as aromatic and aliphatic amines. It was observed that the presence of a nitro group in the *para* position of benzaldehyde significantly hindered the reaction, resulting in lower product yields compared to other substituted aldehydes, even after extended reaction times. The addition of the surfactant at its critical micelle concentration (CMC) led to the formation of micelles in the reaction medium. These micelles created hydrophobic cavities that trapped the lipophilic reactant molecules, thereby enhancing the reaction efficiency and facilitating product formation.

In continuation of studies on the application of ethylenediamine diacetate (EDDA) as a catalyst for organic reactions, Lee et al. [70] (Scheme 48, path B) reported an efficient and environmentally friendly method for the synthesis of 2,3-dihydroquinazolinones using a three-component condensation of isatoic anhydride, anilines, and benzaldehydes in water under reflux for 5–10 h. Importantly, the use of water as a solvent offers environmental benefits and significant rate enhancements. According to the authors, EDDA may act as a Brønsted acid, protonating the carbonyl group of isatoic anhydride and thereby facilitating the nucleophilic attack of the amine, which produced a 2-aminobenzamide derivative. Similarly, EDDA activates benzaldehyde, allowing it to condense with the amino group of anthranilamide to form an imine intermediate, which then undergoes intramolecular cyclization to afford the corresponding lactam. This methodology enables rapid and high-yielding (86–93%) access to a range of 2,3-dihydroquinazolin-4(1*H*)-one derivatives. The reactions worked well with a variety of arylamines bearing either electron-donating or electron-withdrawing groups as well as phenethylamine and 4-phenylbutylamine. Likewise, benzaldehydes with either electron-donating or electron-withdrawing groups on the aromatic ring afforded the desired products in high yields.

In 2016, Azizian and Azimi [71] described a convenient and straightforward route for the synthesis of 2,3-dihydroquinazolin-4(1*H*)-ones via a three-component reaction between isatoic anhydride, various substituted primary amines, and stable benzyl alcohols in place of aldehydes (Scheme 48, path D). Optimal yields and the shortest reaction times (all reactions completed within 3 h) were achieved by performing the process in water under reflux in the presence of equimolar amounts of iodine and potassium carbonate. Under reaction conditions, benzyl alcohols were successfully oxidized in situ to corresponding aldehydes using an environmentally friendly and inexpensive system (I_2/_K_2_CO_3_). A series of a desired products was obtained in excellent yields of up to 93%.

**Scheme 49:**
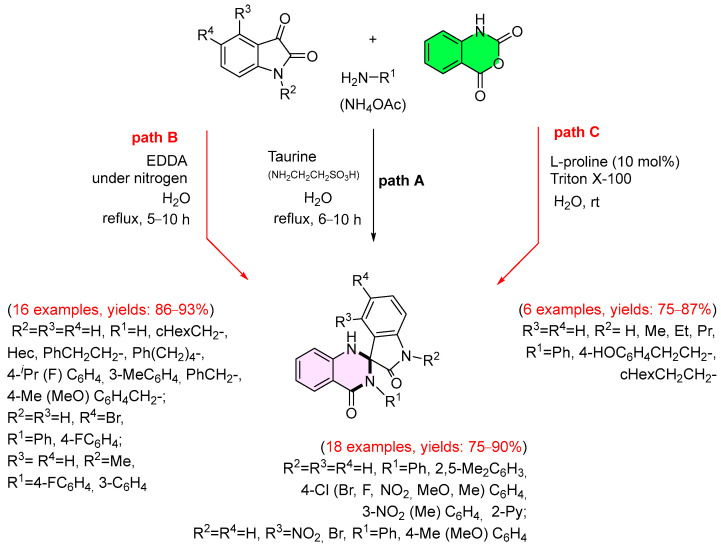
Synthesis of spirooxindole derivatives containing a dihydroquinazolinone ring [68,69,70].

The methodologies described above [68,69,70] were also applied to synthesize spirooxindole derivatives containing a dihydroquinazolinone ring. One-pot, three-component reactions of isatoic anhydride with anilines or ammonium acetate and isatins, used in place of the previously employed aldehydes, were then carried out in water (Scheme 49). The reactions proceeded smoothly and afforded the desired spiro products with excellent yields (75–93%), regardless of the nature of the substitution in the isatin or primary amine moieties and irrespective of the catalysts used.

**Scheme 50:**
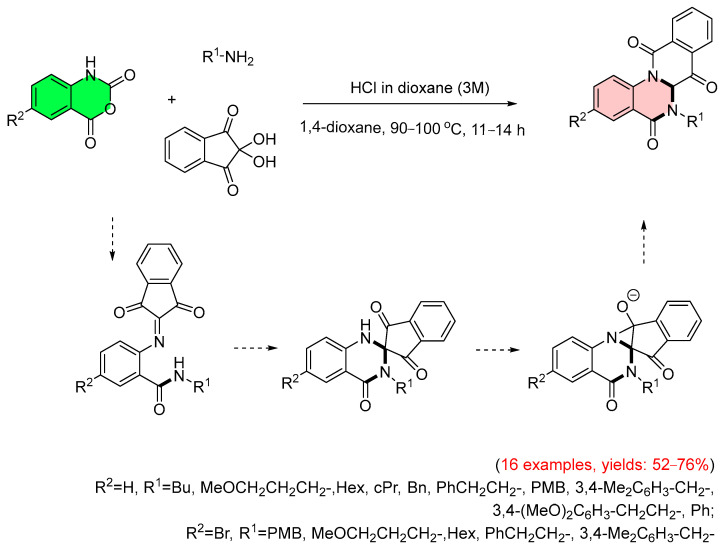
Synthesis of fused quinazolinone-based tetracyclic compounds [72].

The tetracyclic quinazolinone ring system is an important core unit of many biologically active, naturally occurring alkaloids, such as Tryptanthrin, Phaitanthrin C, Ophiuroidine, or (–)-Benzomalvin A and pharmaceuticals [72]. Raghunadh and coworkers [72] developed a straightforward and efficient one-pot strategy for synthesizing fused quinazolinone-based tetracyclic compounds, especially, 6,6a-dihydro-5*H*-isoquinolino[2,3-*a*]quinazoline-5,7,12-trione derivatives (Scheme 50). The key building blocks were isatoic anhydride, a primary amine, and ninhydrin. The reactions were performed in 1,4-dioxane as a solvent with the addition of HCl in dioxane (3 M) solution at 100 °C. The presented procedure yielded a variety of fused tetracyclic quinazolinone derivatives in good-to-excellent yields (52–76%), using both aliphatic and aromatic amines as reactants.

The reaction mechanism proposed by the authors (lower part of Scheme 50) began with a nucleophilic attack of the amine component on the isatoic anhydride, resulting in ring opening and decarboxylation. Subsequent reaction with ninhydrin yielded an imine intermediate, which underwent cyclization to form a spiro compound. In the subsequent step, nucleophilic attack of the amine on the keto group led to the formation of a fused aziridine intermediate, which, after rearrangement, formed the final tetracyclic structure.

## 4. Alternative Synthetic Approaches

**Scheme 51:**
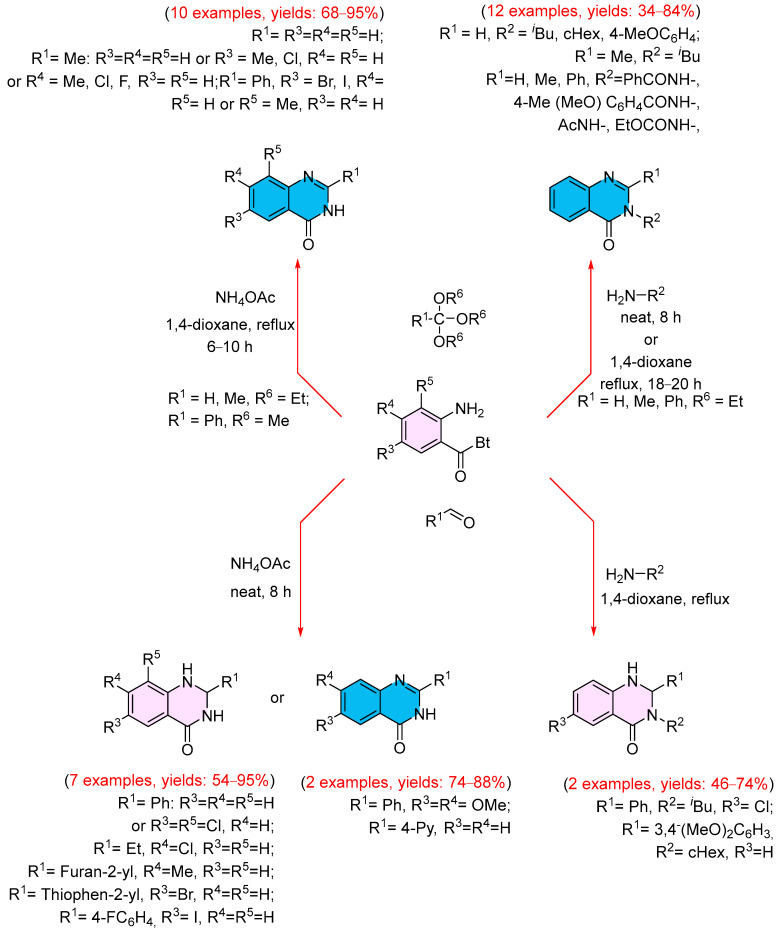
Application of *N*-(2-aminobenzoyl)benzotriazoles in the synthesis of quinazolinones and their 2,3-dihydroderivatives [73,74].

Çelik et al. [73,74] reported a convenient, catalyst-free, and environmentally friendly one-pot method for the synthesis of 2-substituted and 2,3-disubstituted quinazolin-4(3*H*)-ones and 2-substituted and 2,3-disubstituted 2,3-dihydroquinazolin-4(1*H*)-ones using *N*-(2-aminobenzoyl)benzotriazoles as starting materials (Scheme 51).

The procedure involved the use of orthoester in combination with ammonium acetate, primary amine, or hydrazides. The reactions were conducted in 1,4-dioxane under reflux or neat reaction conditions, affording the target products in moderate-to-excellent yields (34–95%). It is proposed that the benzotriazole moiety acts both as an activating group and as a good leaving group, thereby facilitating the ring closure.

**Scheme 52:**
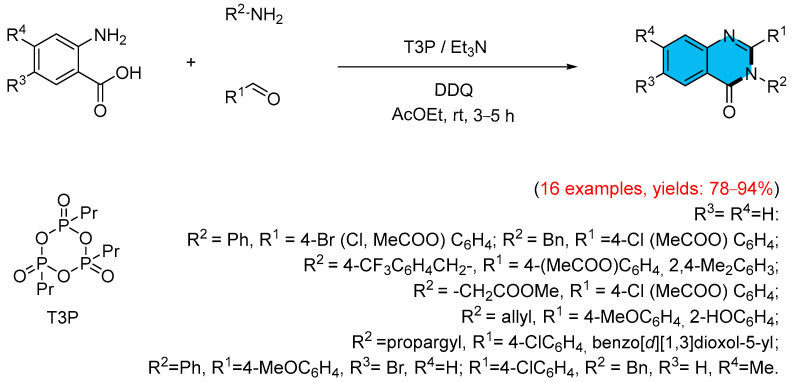
Application of T3P in the synthesis 2,3-disubstituted quinazolinones [75].

Mantelingu et al. [75] described an efficient methodology for the synthesis of various 2,3-disubstituted quinazolin-4(3*H*)-ones via a one-pot three-component reaction starting from anthranilic acids, using T3P (propylphosphonic anhydride) as the catalyst and DDQ (2,3-dichloro-5,6-dicyano-1,4-benzoquinone) as the oxidant (Scheme 52). Initially employed as a peptide coupling agent and water scavenger with low toxicity, T3P has also proven effective in rearrangement reactions, heterocyclic synthesis, and as an active catalytic system. The procedure involved the in situ coupling of various amines (anilines, benzylic amines, allylamines, amino esters) with an activated-by-T3P 2-aminobenzoic acid and its 5-bromo- and 4-methyl derivatives, followed by the condensation of the resulting amide intermediate with aromatic aldehydes in the presence of T3P to form an imine intermediate. The next step involved intramolecular cycloaddition of imine, protonated by P,P′,P″-tripropyl triphosphonic acid, yielding the 2,3-dihydriquinazolinone as a key intermediate. The further dehydrogenation mediated by DDQ led to the formation of 2,3-disubstituted quinazolin-4(3*H*)-ones. In all cases, regardless of the type of substrates used, the target products were obtained in excellent yields (78–94%). The main advantages of this protocol are mild reaction conditions—the reaction proceeds at room temperature, has a short reaction time, and allows for easy isolation of products.

**Scheme 53:**
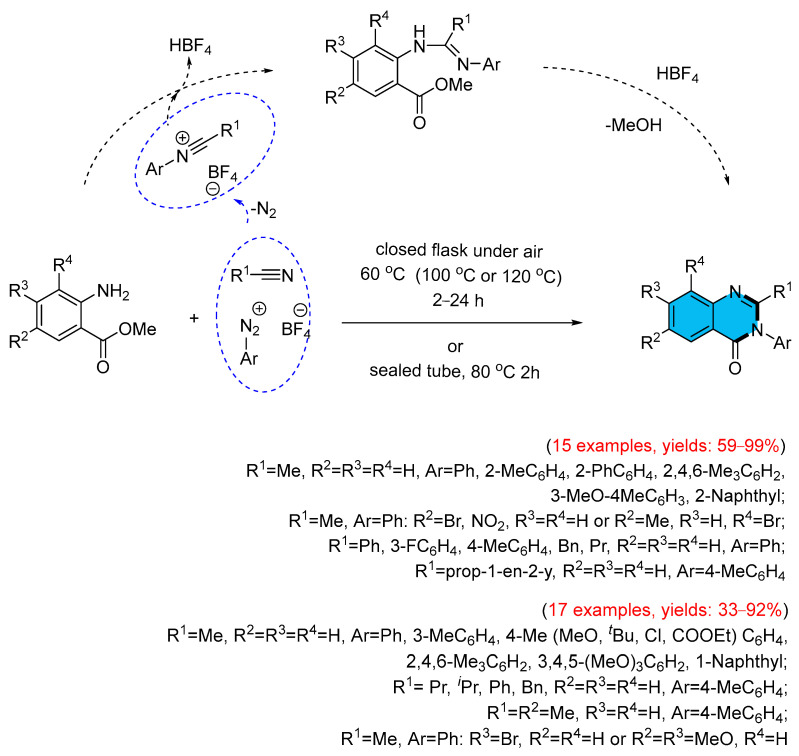
Application of aryldiazonium tetrafluoroborate in the synthesis of 3-arylquinazolinones [76,78].

In 2024, Youn et al. [76] and, earlier, Liu et al. [77,78] reported the utilization of aryldiazonium tetrafluoroborate as a precursor for the synthesis of 3-arylquinazolin-4(3*H*)-ones (Scheme 53). The ease of preparation from a wide range of commercially available anilines, combined with the excellent leaving group ability of N_2_ and the inherent electrophilicity of aryldiazonium salts, makes them an attractive alternative to aromatic halides. Their synthetic applications include, among others, reactions with nitriles and the in situ generation of *N*-arylnitrilium salts as intermediates, which can subsequently react with various amino and carbon nucleophiles, such as methyl anthranilate, in a cascade reaction under metal-free conditions to afford quinazolinone derivatives. This annulation protocol proceeded well with various aryldiazonium salts bearing substitutions such as 3-Me, 4-Me, 4-*^t^*Bu, and 4-OMe, as well as 4-Cl and 4-COOEt. Moreover, the presence of sterically demanding substituents in the aryldiazonium salts, including 2,4,6-trimethyl and 3,4,5-trimethoxy groups, was well tolerated, producing the multi-substituted quinazolinones in moderate-to-good yields. Furthermore, various readily available aliphatic, aromatic, and benzylic nitriles, as well as methyl anthranilates bearing diverse substituents such as 5-Me, 5-Br, 5-NO_2_, 4-Br, 4,5-dimethoxy, and 3-Br-5-Me groups, smoothly participated in the transformation, affording the corresponding quinazolin-4(3*H*)-ones. In contrast, malononitrile and 3-bromopropionitrile failed to yield the desired products. Importantly, the reaction required anhydrous conditions. Otherwise, hydrolysis of the nitrilium ion could presumably occur, forming the corresponding amide as the major product [78]. As demonstrated by Liu et al. [78], in the case of 2-phenyl substituted aryldiazonium tetrafluoroborate, the *N*-arylnitrilium salt, generated from the reaction with acetonitrile, with the loss of N_2_, preferentially reacted with methyl anthranilate in an intermolecular amination process to furnish 3-aryl-2-methylquinazolinone. This pathway was favored over the competing intramolecular arylation, which would otherwise lead to the formation of 6-methylphenanthridine. As shown in the Scheme 53, the reaction of the *N*-arylnitrilium salt with methyl anthranilate occurred via the formation of an *N*,*N′*-diarylamidine intermediate, which subsequently underwent an intramolecular nucleophilic substitution and annulation, yielding quinazolinone in an excellent yield. The HBF_4_ generated in situ likely acted as a catalyst, activating the ester group via coordination and thereby facilitating the cyclization process [76].

**Scheme 54:**
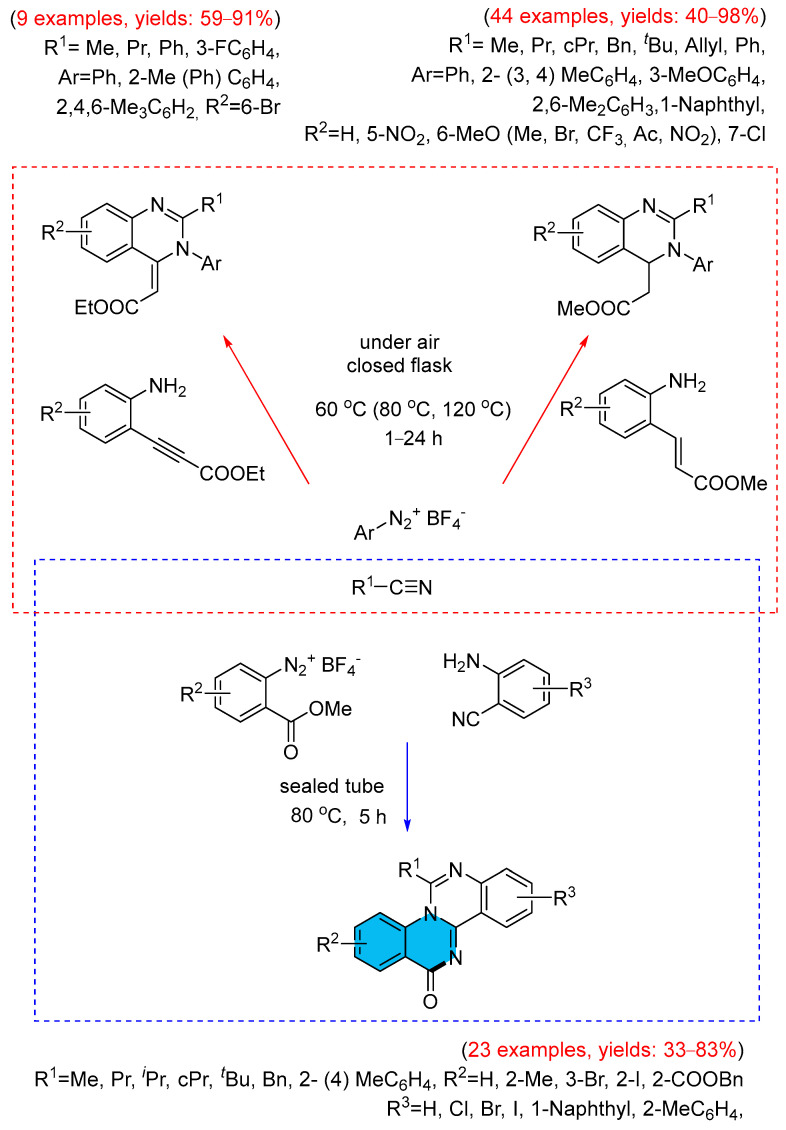
Synthesis of 3-aryl-4-carbonylmethyl (carbonylmethylene) 3,4-dihydroquinazolines and quinazolino-quinazolinone derivatives [76,77].

This methodology was also applied in the synthesis of 3-aryl-4-carbonylmethyl (or carbonylmethylene) 3,4-dihydroquinazolines from 2-alkenyl and 2-alkynyl-substituted anilines, nitriles, and aryldiazonium salts [76], as well as in the preparation of substituted quinazolino-quinazolinone derivatives (13*H*-quinazolino[3,4-a]quinazolin-13-ones) [77] (Scheme 54). The latter compounds were obtained from *o*-ester-substituted aryldiazonium salts, nitriles, and 2-cyanoanilines via the in situ generation of *N-*arylnitrilium intermediates. These intermediates were formed by the reaction of 2-(methoxycarbonyl)aryldiazonium salts and nitriles, followed by tandem amination, cascade cyclization, and amidation steps to afford the fused products.

**Scheme 55:**
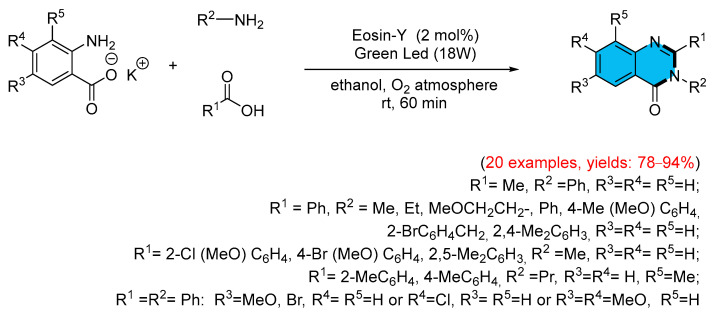
Application of Eosin-Y as the photocatalyst in the synthesis of 2,3-disubstituted quinazolinones [79].

In 2024, Singh et al. [79] developed a one-pot, environmentally friendly, and metal-free synthetic method for the preparation of biologically potent quinazolinone derivatives (Scheme 55). The method involved a multicomponent reaction between the potassium salts of various substituted or unsubstituted anthranilic acids, an aromatic carboxylic acid, and an aliphatic or aromatic amine, using Eosin-Y as the photocatalyst. The reactions were carried out in ethanol, which proved to be the most optimal solvent among those tested (MeOH, water, MeCN, or DCM), under an O_2_ atmosphere at room temperature and irradiated with a green LED (18W) as the energy source. By optimizing the reaction conditions, it was found that both the photocatalyst and visible light were essential for achieving good yields and also supported the photocatalytic reaction mechanism. Additionally, the process eliminates the need for toxic metals, high temperatures, or hazardous reagents. Overall, the protocol proved to be compatible with a wide range of substrates containing both electron-withdrawing and electron-donating groups on the benzene ring, yielding quinazolinones in excellent yields, up to 94%. The proposed mechanism involved a photoexcited Eosin-Y-mediated single electron transfer (SET) process, supported by mechanistic studies, confirming the generation of reactive oxygen species.

**Scheme 56:**
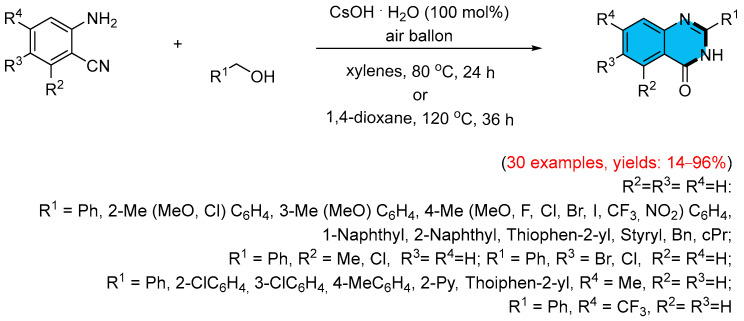
CsOH-mediated aerobic oxidative annulation of *o*-aminobenzonitriles and alcohols [80].

A simple and practical one-pot approach for the efficient synthesis of a wide range of substituted quinazolinones, based on a base-catalyzed aerobic oxidative annulation of 2-aminobenzonitriles with alcohols, was developed by Xu and co-workers [80]. A key role in this transformation was played by cesium hydroxide, which catalyzed both the nitrile hydration and the aerobic oxidation of alcohols to aldehydes (Scheme 56). As demonstrated, no reaction occurred in the absence of CsOH, and the reaction was less effective without adequate air, indicating that both the base and the amount of air were crucial for the reaction. Moreover, air was found to be more effective than pure O_2_, generating water as the only byproduct. The optimal conditions for synthesizing the desired compounds were found to be 100 mol% CsOH in xylenes under reflux at 80 °C for 24 h. The optimized reaction conditions enabled the smooth synthesis of quinazolinone derivatives using a wide variety of benzylic alcohols with various *para*-, *meta*-, and *ortho*-substituted electron-donating and electron-withdrawing groups, as well as both electron-rich and electron-deficient *o*-aminobenzonitriles. Generally, the electron-rich benzyl alcohols furnished good-to-high yields of the products. In contrast, the reactions involving electron-deficient benzylic alcohols appeared to be less efficient; notably, *para*-nitrobenzyl alcohol did not yield any target product under a variety of conditions. 2-Pyridylmethanol and 2-thienylmethanol yielded moderate results under standard conditions, and higher yields were achieved under modified conditions with 1,4-dioxane as the solvent. Among aliphatic alcohols, only cyclopropylmethanol was found to be reactive.

**Scheme 57:**
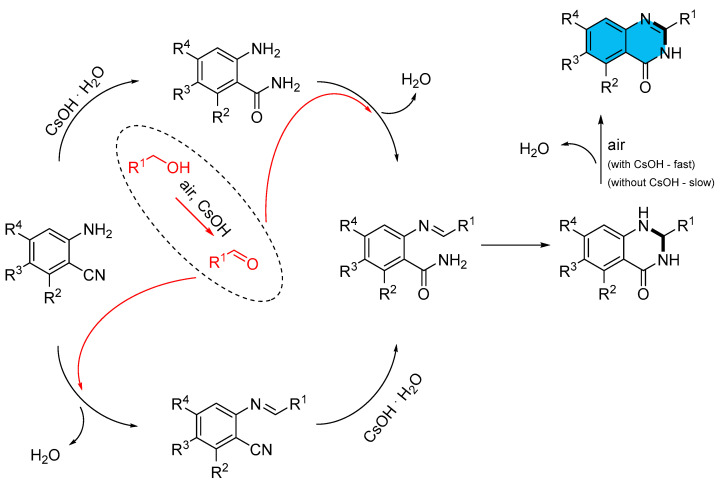
Suggested mechanism for the CsOH-mediated synthesis of quinazolinones [80].

Based on the results of a series of control experiments, the authors proposed that the reaction begins with the CsOH-mediated hydration of *o*-aminobenzonitrile followed by aerobic oxidation of the alcohol to the corresponding *o*-aminobenzamide and aldehyde. As shown in Scheme 57, both *o*-aminoarylnitrile and *o*-aminobenzamide can react with an aldehyde to form *o*-iminebenzonitrile and *o*-iminebenzamide intermediates, the former of which can undergo hydration to an amide. Subsequent oxidative annulation leads to the formation of quinazolinone via a 2,3-dihydroquinazolinone intermediate.

**Scheme 58:**
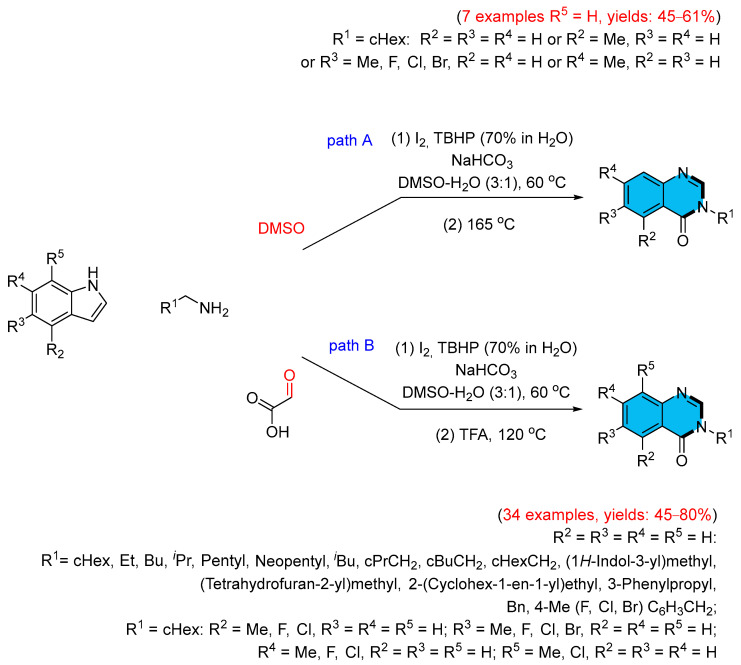
Iodine-catalyzed synthesis of 2-substituted quinazolinones from indoles and amines [81].

Zhang et al. [81] developed a strategy for synthesizing 2-substituted quinazolin-4(3*H*)-ones via an iodine-catalyzed, multicomponent domino reaction. This strategy used indoles, glyoxylic acid, and amines as the starting materials (Scheme 58, path B). Reaction optimization revealed that using Cs_2_CO_3_ as the base, *tert*-butyl hydroperoxide (TBHP) as the oxidant, and a DMSO-H_2_O (3:1) solvent system was most effective, affording the target products in moderate-to-good yields (45–80%), depending on substrate structure. Interestingly, performing the reaction in dry DMSO significantly reduced the yield, confirming the essential role of water in promoting reaction homogeneity. In addition, the synthetic protocol was also successfully executed without glyoxylic acid—using only DMSO and H_2_O—through minor modifications to the reaction temperature (Scheme 58, path A).

Indoles bearing Me, I, F, Br, or Cl substituents on the phenyl ring were well tolerated under the optimized conditions. Their reactions with cyclohexylamine and glyoxylic acid afforded the corresponding lactams in a 53–75% yield. Moreover, it was observed that substituents on the indole aromatic ring, particularly Me at the C7 position, exhibited steric effects. Additionally, the reaction showed broad tolerance toward diverse amines. Both alkyl amines of varying chain lengths (e.g., ethyl, butyl, pentyl, cetyl) and sterically hindered amines (e.g., neopentylamine, isobutylamine, cycloheptanamine) were compatible. Furthermore, benzylamines bearing either electron-donating or electron-withdrawing groups on the aromatic ring, as well as tetrahydrofurfurylamine and tryptamine, also participated effectively in the transformation.

**Scheme 59:**
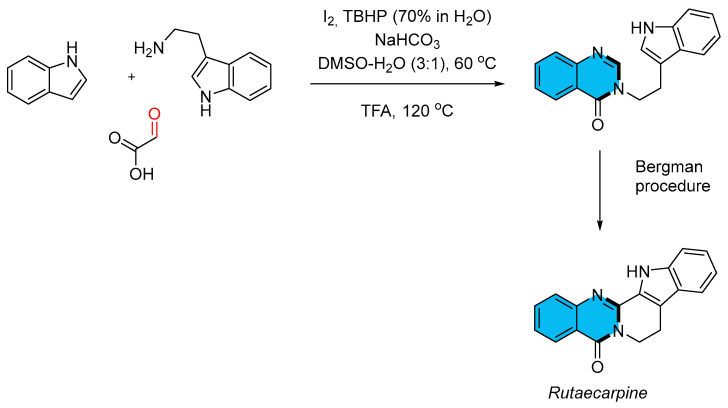
Synthesis of Rutaecarpine [81].

The synthetic potential of this method was demonstrated in the two-step synthesis of the alkaloid Rutaecarpine, employing indole, tryptamine, and glyoxylic acid as reactants in a single domino procedure, followed by Bergman’s protocol. Additionally, the procedure was successfully scaled-up to the gram scale (Scheme 59).

**Scheme 60:**
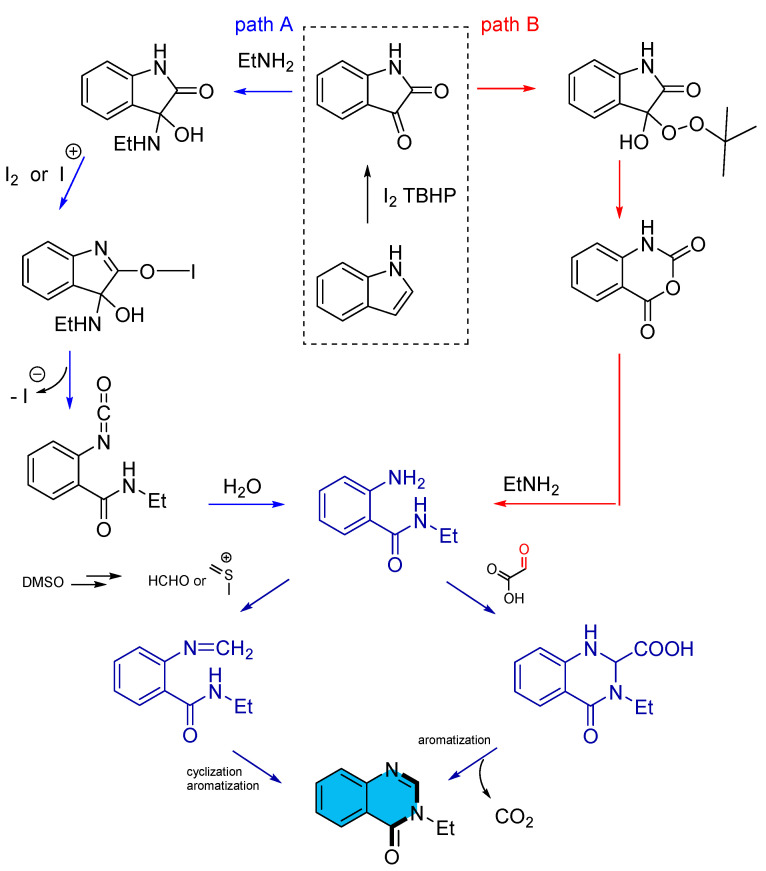
Suggested mechanism for the iodine-catalyzed synthesis of 2-substituted quinazolinones from indoles [81].

According to a comprehensive mechanistic investigation (Scheme 60), the reaction begins with the oxidation of indole to isatin via the I_2/_TBHP-mediated system. It can be assumed that the oxidative cleavage of the C=C in indole proceeds via a radical-mediated pathway, as evidenced by the fact that the addition of TEMPO, a radical scavenger, inhibits the reaction. The subsequent formation of an anthranilamide derivative from isatin can occur via two possible pathways. In the first pathway, isatin is transformed into isatoic anhydride through Baeyer–Villiger oxidation, as a result of the action of the TBHP/NaHCO_3_ system. This isatoic anhydride then condenses with amines to yield a 2-aminobenzamide intermediate. Alternatively, in the second pathway, isatin may undergo the I^+^-promoted condensation with an amine, leading to the rapid formation of an isocyanate intermediate, which then hydrolyzes to the same anthranilamide derivative. Further reaction with glyoxylic acid or DMSO acting as a source of the carbon atom ultimately yields quinazolinone.

**Scheme 61:**
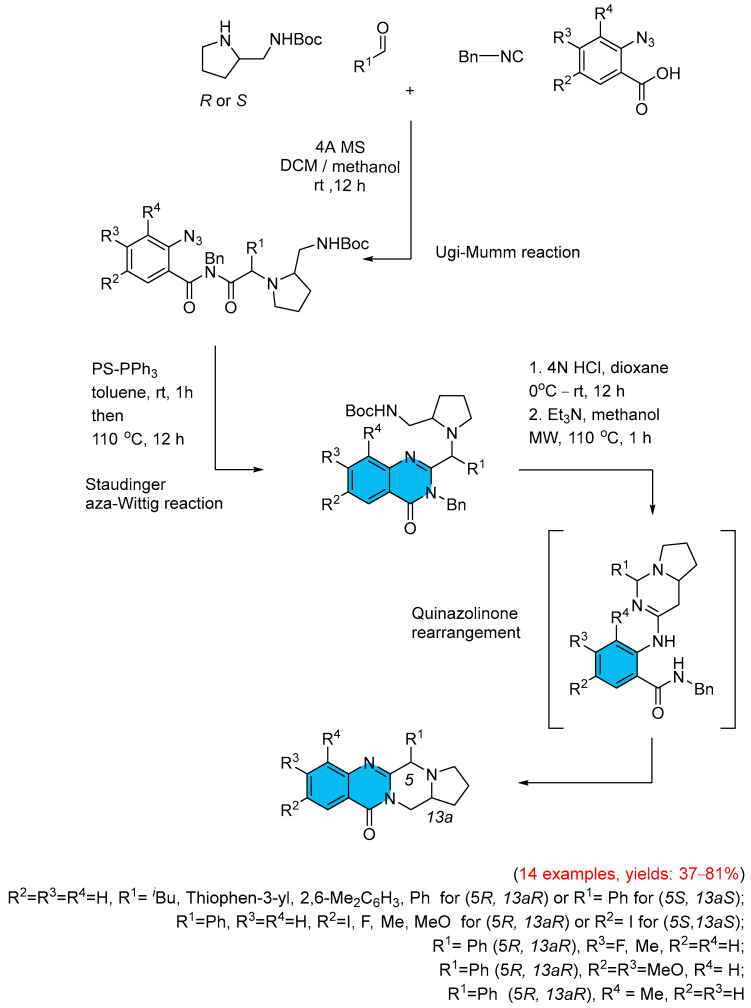
Synthesis of pyrrolopyrazinoquinazolinones [82].

Pyrrolopyrazinoquinazolinones are a group of heterocyclic compounds with promising pharmaceutical potential. An interesting approach to the synthesis of enantiopure pyrrolopyrazinoquinazolinones, based on the four-component Ugi–Mumm–Staudinger–*aza*-Wittig sequence followed by tandem quinazolinone rearrangement to tautomeric *Z*-benzamidines and their intramolecular cyclization to the title compounds was reported in 2021 by Golden et al. [82] (Scheme 61).

The inspiration for this research stemmed from an earlier observation of a regiospecific rearrangement of quinazolinones to (*E*)-benzamidines, which employed secondary amines as part of the quinazolinone skeleton [83]. In 2019, the same authors [83] reported an efficient one-pot, multicomponent strategy leading to a diverse array of C3-functionalized (*E*)-arylamidines bearing either *N*-alkyl or *N*-aryl amide groups via the in situ formation of the quinazolinone scaffold from an imide intermediate resulting from the Ugi–Mumm rearrangement under the Staudinger–*aza*-Wittig conditions, followed by its rearrangement after deprotection and microwave irradiation in Et_3_N/MeOH.

The synthesis involved *N*-Boc derivative of *N*,*N*′-dimethylethylenediamine, various isocyanides (such as isopropyl isocyanide, cyclohexyl isocyanide, or 4-methoxyphenyl isocyanide), a broad array of aldehydes, including formaldehyde, and 2-azidobenzoic acid, along with its 4-Me, 5-Me, 5-NO_2_, 4-F, 5-I, and 5-F derivatives, affording the target amidines in moderate-to-excellent yield (31–88%) [83]. On the other hand, the four-component Ugi–Mumm–Staudinger–*aza*-Wittig sequence proved to be an excellent method for the preparation of 2-{[2-((*tert*-butoxycarbonyl)(methyl)amino)ethyl(methyl)amino]methyl} derivatives of 3-alkyl or 3-aryl substituted quinazoline-4(3*H*)-ones, affording the products in 60–89% yields.

The use of *tert*-butyl (*R*)-(pyrrolidin-2-ylmethyl)carbamate or *tert*-butyl (*S*)-(pyrrolidin-2-ylmethyl)carbamate in place of an achiral *N*-Boc protected secondary amines in combination with benzaldehyde, 2-azidobenzoic acid, and benzyl isocyanide under previously reported conditions delivered C5-phenyl-substituted pyrroloquinazolinones as mixtures corresponding two diastereomers, (5*R*,13a*R*) and (5*S*,13a*R*), with a diastereomeric ratio of 89:11 and an overall yield of 79%, and (5*S*,13a*S*) and (5*R*,13a*S*), with an 86:14 d.r., in a 76% yield, respectively [82]. Hence, all four stereoisomers of the target compounds could be obtained and isolated through a single achiral separation of diastereomers from two independent reactions. Extending the method to other substituted 2-azidobenzoic acid, such as 3-Me, 4-F (Me), 5-F (I, Me), as well as replacement of benzaldehyde with other aldehydes, including pivaldehyde, 2,6- dimethylbenzaldehyde, 3-thiophenecarbaldehyde, or isobutyraldehyde, ultimately afforded 15 diastereomeric pyrazinoquinazolinone pairs in an up to 83% overall yield and with diastereomeric ratios of up to 89:11.

## 5. Conclusions

Quinazolin-4(3*H*)-one and its derivatives are pharmacologically active nitrogen-containing heterocycles that have attracted considerable interest in the field of medicinal chemistry. Therefore, the development of novel, effective, and greener strategies for con-structing the quinazolinone scaffold remains a significant scientific challenge, particularly in the context of their potential applications in the pharmaceutical industry.

The development of new synthesis methods involves not only the design of new reagents or catalysts but also the application of alternative activation techniques to conventional heating, such as microwave irradiation, ultrasound, photocatalysis, and mechanochemical activation. These aspects are also partially addressed in this review

The main aim of this review is to summarize a wide range of innovative and efficient methods for constructing quinazolinone skeleton based on one-pot multicomponent reactions. MCRs serve as a powerful tool for generating libraries of highly functionalized compounds, often from readily available and inexpensive starting materials.

This review focuses on the discussion of reaction conditions, substrate scope, and development of new reaction types and catalytic systems. In addition, the most important mechanistic aspects of the reported methods are highlighted.

An equally important aspect discussed in the article is the environmental friend-liness of the applied methodologies, including the use of water as a solvent, the avoid-ance of toxic reagents, simple product separation, and catalyst recycling.

Recent advances in the development of novel methodologies for the preparation of quinazolinone derivatives include, for example, a solvent- and oxidant-free mechanocatalyzed protocol reported by Sharma et al. [84] for the reaction of various substituted 2-aminobenzamides with 2-oxo-2-phenylacetic acids under ball-milling at room temperature. Similarly, Maiti et al. [85] described the reaction of substituted 2-aminobenzamides with dimethyl sulfoxide used as a carbon source in the presence of H_2_O_2_ as an effective and green oxidant. In addition, Gong et al. [86] developed a metal- and oxidant-free strategy for the one-pot synthesis of 2,3-disubstituted quinazolin-4(3*H*)-ones by combining acid-catalyzed cyclization and direct anodic oxidation, using 2-aminobenzamides and aldehydes as starting materials.

In conclusion, it can be said that concern for the environment will play a key role in shaping the directions of future research in the field of quinazolinone synthesis.

## Data Availability

No new data were created or analyzed in this study.

## Abbreviations and Acronyms

The following abbreviations are used in this manuscript:

Ac	acetyl
AcOH	acetic acid
Bn	benzyl
Boc	*tert*-butoxycarbonyl
BuPAd_2_	di-1-adamantyl-*n*-butylphosphine
Bu	*n*-butyl
*^i^*Bu	isobutyl
*^t^*Bu	*tert*-butyl
cBu	cyclobutyl
NaO*^t^*Bu	sodium *tert*-butoxide
Bt	benzotriazol-1-yl
Cu(OAc)_2_	copper(II) acetate
Cu(OTf)_2_	copper(II) trifluoromethanesulfonate, copper(II) triflate
CMC	critical micelle concentration
DCE	1,2-dichloroethane
DIPEA	*N*,*N*-diisopropylethylamine
DMAc	*N*,*N*-dimethylacetamide
DBU	1,8-diazabicyclo[5.4.0]undec-7-ene
DABCO	1,4-diazabicyclo[2.2.2]octane
DDQ	2,3-dichloro-5,6-dicyano-1,4-benzoquinone
DMF	dimethylformamide
DMSO	dimethyl sulfoxide
DPPP	1,3- bis(diphenylphosphino)propane
DTBP	di-*tert*-butyl peroxide
EDDA	ethylenediamine diacetate
GO	graphene oxide
rGO	reduced GO
N-rGO	nitrogen-doped reduced graphene oxide
LiHMDS, LHMDS	lithium hexamethyldisilazane, lithium bis(trimethylsilyl)amide
Me	methyl
MeO	methoxy
MeS	methylsulfanyl
MeCN	acetonitrile
NMe_2_	dimethylamino
Ph	phenyl
PPh_3_	triphenylphosphine
PCy_3_	tricyclohexylphosphine
Xantphos	4,5-bis(diphenylphosphino)-9,9-dimethylxanthene
DPPB	1,4-bis(diphenylphosphino)butane
BINAP	2,2′-bis(diphenylphosphino)-1,1′-binaphthyl
P4VP	poly(4-vinylpyridine)
[Cp*IrCl_2_]_2_	pentamethylcyclopentadienyliridium(III) chloride, dimer
DPPF	1,1′-ferrocenediyl-bis(diphenylphosphine)
DCM	dimethylchloromethane
Hex	*n*-hexyl
cHex	cyclohexyl
HBT	*N*-hydroxybenzotriazole
Pentyl	*n*-pentyl
Neopentyl	2,2-dimethylpropyl
PEG-400	poly(ethylene glycol) 400
PMB	*para*-methoxybenzyl
Pr	*n*-propyl
*^i^*Pr	isopropyl
cPr	cyclopropyl
2-Py	2-pyridyl
3-Py	3-pyridyl
4-Py	4-pyridyl
Pd(OAc)_2_	palladium(II) acetate
PdCl_2_	palladium(II) chloride
Pd(TFA)_2_	palladium(II) trifluoroacetate
Pd_2_(dba)_3_ CHCl_3_	tris(dibenzylideneacetone)dipalladium(0)-chloroform adduct
Pd(PPh_3_)_2_Cl_2_	bis(triphenylphosphine)palladium(II) dichloride
PTSA	*para*-toluenesulfonic acid
T3P	propylphosphonic anhydride
TPPMS	sodium diphenylphosphinobenzene-3-sulfonate
TBHP	*tert*-butyl hydroperoxide
TFA	trifluoroacetic acid
TEMPO	2,2,6,6-tetramethyl-1-piperidinyloxy
TEOA	triethanolamine
THF	tetrahydrofuran
THF-2-yl	tetrahydrofuran-2-yl
Tol	*p*-tolyl
2-MeTHF	2-methyltetrahydrofuran
TMSN_3_	trimethylsilyl azide
Ts	tosyl (*p*-CH_3_C_6_H_4_SO_2_)
TsN_3_	*p*-toluenesulfonyl azide, tosyl azide
TsNH_2_	*p*-toluenesulfonamide
Triton X-100	polyethylene glycol *tert*-octylphenyl ether
US	ultrasound irradiation
MW	microwave irradiation
